# The dynamics of gas-bubble formation at saturated conditions in porous media flow

**DOI:** 10.1038/s41598-020-69506-w

**Published:** 2020-08-06

**Authors:** K. Alex Chang, W. Brent Lindquist

**Affiliations:** 1 Department of Applied Mathematics, National Pingtung University, Pingtung, Taiwan ROC; 2 Departments of Mathematics and Statistics and Geosciences, Texas Tech University, Lubbock, TX USA

**Keywords:** Carbon capture and storage, Petrol, Applied mathematics

## Abstract

We investigate the stability of gas bubbles formed at saturated (bubble-point) conditions during two-component ($\hbox {CO}_2$, $\hbox {H}_2$O), two-phase (gas, liquid) flow by developing and analyzing a $2\times 2$ dynamical system describing flow through a single pore to study the dynamics of gas bubble formation and evolution. Our analysis indicates that three regimes occur at conditions pertinent to petroleum reservoirs. These regimes correspond to a critical point changing type from an unstable node to an unstable spiral and then to a stable spiral as flow rates increase. In the stable spiral case gas bubbles will achieve a steady-state finite size only if they form within the attractor region of the stable spiral. Otherwise, all gas bubbles that form undergo, possibly oscillatory, growth and then dissolve completely. Under steady flow conditions, this formation and dissolution repeats cyclically.

## Introduction

Compositional flow involving a dissolved gas is of importance in many areas, including oil reservoir production^3, 14, 22^, pipeline transport^12, 19, 25, 28^, $\hbox {CO}_2$ sequestration^6, 27^, and the disposal of radioactive waste^4, 5^. Such flow involves the inherent possibility of creation of a gas phase and its subsequent transport. Excluding specific tertiary recovery practices such as $\hbox {CO}_2$ foam flooding^24^, keeping potential gas components dissolved in fluid phases is important for efficient extraction in reservoirs; the presence of gas bubbles and the resultant fluid–gas menisci complicates flow and can compete with fluid movement. The ability to prevent bubble formation through control of formation pressure or flow rates is therefore important for extraction efficiency.

Once formed in a porous medium, the gas phase is typically non-wetting. During imbibition (displacement by a wetting phase) a non-wetting phase can either exit pore space completely under piston-like displacement, or a fraction of it may become trapped in the form of one or more bubbles by a process known as snap-off. The snap-off process is strongly dependent on pore-geometry, wettability, viscosity and interfacial-tension conditions^26^. While snap-off is an important process in trapping the non-wetting phase, the process requires a starting condition in which both phases—non-wetting and wetting—are present.

In this study, we focus instead on the process by which a gas phase comes out of a saturated solution—forming bubbles. We therefore directly address the transition between single- and two-phase flow and the initial dynamics of those gas bubbles that do form. We are consequently studying flow conditions that would occur before sufficient (local) volumes of non-wetting phase have formed to begin any process of snap-off.

A challenge to the numerical simulation of compositional flow in porous media is the change in the system of equations that accompanies the appearance or disappearance of the gas phase. This difficulty has been addressed in several computational approaches^1, 2, 6–8, 10, 21^. In our computations of two-phase, two-component ($\hbox {H}_2\hbox {O}$, $\hbox {CO}_2$) flow in a 3D pore network^6^, we noted the periodic appearance and dissolution of the gas phase in certain pores. Intensive evaluation of our algorithms led us to conclude that this observed cyclic phenomenon was not numerical in origin. In reviewing the literature on gas transport in porous media at reservoir scales^13^, in micromodel studies^23^, specific studies on gas bubble formation^17^, and mathematical studies of gas phase disappearance in water-hydrogen systems^15^, we have been unable to find any mention of this periodic phenomenon. We have therefore pursued a mathematical investigation. In this article, we extract a $2\times 2$ dynamical system from the mathematical model upon which our computations in Chang and Lindquist^6^ were based in order to study the dynamics of gas bubble formation and evolution and the mechanics of this phase-cycling phenomenon. We summarize the mathematical model and derive the dynamical system. We analyze the direction fields for this non-linear system, demonstrate the existence of critical points, and study solution trajectories. To support our analysis of the dynamical system, we obtain numerical solutions and conclude with a discussion of our results.

## The mathematical model and the dynamical system

Here we summarize the model^6^ in the context of flow through a single pore and develop the dynamical system.

### The physical model

**Figure 1:**
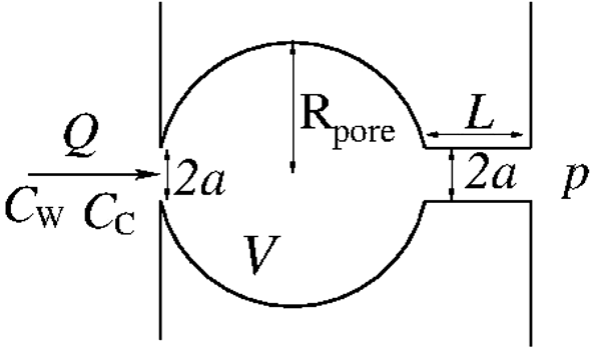
A cross section of the axially symmetric 3D geometry. The arrow indicating flow direction is placed on the axis of symmetry.

Consider a 3D, horizontal, axially symmetric network model consisting of a single spherical pore of radius ${\mathrm R}_{\mathrm{pore}}$ and volume *V* (Fig. 1). The flow through the network is from left to right at the constant volumetric rate *Q*. The inflow solution is a liquid phase consisting of water with dissolved $\hbox {CO}_2$; the concentrations of $\hbox {H}_2$O and $\hbox {CO}_2$ in the solution are $C_{\mathrm{ W }}$ and $C_{\mathrm{ C }}$, respectively.

**Figure 2:**
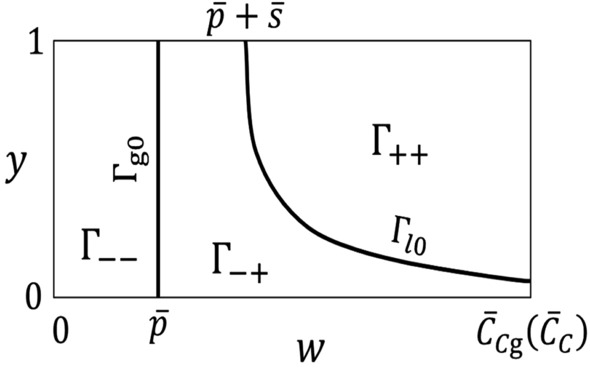
The phase space is divided into three regions, ${\Gamma }_{++}$, ${\Gamma }_{-+}$ and ${\Gamma }_{--}$, by the curves ${\Gamma }_{{{ l}0}}$ and ${\Gamma }_{{\mathrm{g}{0}}}$.

The pore is initially filled with pure water. At the outlet, the pore is connected to a large reservoir of pure water held at pressure *p*. The length of the outlet channel is *L* and the radius of the cross section of the inlet and outlet channel is *a*. The system is held at constant temperature *T*.

### The mathematical model

Under single-phase flow conditions (flow remains under-saturated with dissolved $\hbox {CO}_2$) the fluid transport is governed by the equations

1 $$\begin{aligned} V\frac{dC_{\mathrm{ W }}}{dt}=QC_{\mathrm{ W }}-C_{\mathrm{ W }} {\Lambda }_l (p_{l}-p), \qquad V\frac{dC_{\mathrm{ C }{ l}}}{dt}=QC_{\mathrm{ C }}-C_{\mathrm{ C }{ l}} {\Lambda }_l (p_{l}-p), \end{aligned}$$

where $C_{\mathrm{ C }l}$ and $p_{l}$ are, respectively, the concentration of $\hbox {CO}_2$ and the pressure of the liquid phase in the pore and ${\Lambda }_l$ is the conductivity of the liquid phase from the pore to the outlet. The liquid phase is assumed incompressible with $\hbox {H}_2$O remaining the dominant species; therefore the concentration of water in the liquid phase is assumed to be the constant value $C_{\tiny {\text{ W }}}=1/18$ mol cm$^{-3}$. Under these assumptions, the equations in (1) simplify to

2 $$\begin{aligned} p_{l}^{\mathrm{sp}} = Q/{\Lambda }_l + p,\quad C_{\mathrm{ C }{ l}}(t) = C_{\mathrm{ C }}(1 - e^{-Qt/V}), \end{aligned}$$

which provide the single-phase liquid pressure of the water and the $\hbox {CO}_2$ concentration in the pore.

A gas phase is generated in the pore when the concentration of $\hbox {CO}_2$ exceeds the solubility (see equation (17) of Chang and Lindquist^6^) of dissolved $\hbox {CO}_2$ in the liquid phase at the pressure $p_{l}^{\mathrm{sp}}$, and the resulting two-phase flow follows the system below (these are, respectively, Eqs. (1), (9), (3), (4), (14) and (15) of Chang and Lindquist^6^ applied to our single pore geometry):

3a $$\begin{aligned}&s_{l} + s_{\mathrm{g}}=1, \end{aligned}$$

3b $$\begin{aligned}&p_{\mathrm{g}}=p_{l} + p_{{ c}}, \end{aligned}$$

4a $$\begin{aligned}&V\dfrac{dm_{\mathrm{H}}}{dt}=\left\{ \begin{array}{ll} QC_{\mathrm{ W }}- Q_{{ l}} C_{\mathrm{ W }}-Q_{\mathrm{g}} C_{\mathrm{Hg}}, &\quad {}p_{\mathrm{g}}>p,\\ QC_{\mathrm{ W }}-Q_{{ l}} C_{\mathrm{ W }}, &\quad {}p \ge p_{\mathrm{g}},\end{array}\right. \end{aligned}$$

4b $$\begin{aligned}&V\dfrac{dm_{\mathrm{C}}}{dt}=\left\{ \begin{array}{ll} QC_{\mathrm{C}}- Q_{{ l}} C_{\mathrm{C}{} { l}}-Q_{{ g }} C_{\mathrm{Cg}} ,&\quad {}p_{\mathrm{g}}>p_{{ l}}>p ,\\ QC_{\mathrm{C}}-Q_{\mathrm{g}} C_{\mathrm{Cg}}, &\quad {}p_{\mathrm{g}}> p \ge p_{{ l}}, \\ QC_{\mathrm{C}} , &\quad {}p \ge p_{\mathrm{g}} > p_{{ l}},\end{array}\right. \end{aligned}$$

5a $$\begin{aligned}\mu _{\mathrm{H} l}^\ominus + RT \ln \left( \dfrac{C_{\mathrm{ W }}}{C_{\mathrm{ W }}+C_{\mathrm{C}{ l}}} \right) =\mu _{\mathrm{Hg}}^\ominus + RT \ln \left( \dfrac{C_{\mathrm{Hg}}RT}{p_{\mathrm{H}}^*} \right) , \end{aligned}$$

5b $$\begin{aligned}\mu _{\mathrm{C}{} { l}}^\ominus + RT \ln \left( \dfrac{C_{\mathrm{C}{ l}}}{C_{\mathrm{ W }}+C_{\mathrm{C}{} { l}}} \right) =\mu _{\mathrm{Cg}}^\ominus + RT \ln \left( \dfrac{C_{\mathrm{Cg}}RT}{p_{\mathrm{C}}^*} \right) . \end{aligned}$$

In this system, $s_{{ l}}$ and $s_{\mathrm{g}}$ are the saturations of the liquid and gas phases; $p_{{ l}}$ and $p_{\mathrm{g}}$ are the pressures in the liquid and gas phase in the pore; $C_{\mathrm{Hg}}$ is the gas phase $\hbox {H}_2$O concentration; $C_{\mathrm{C}{} { l}}$ and $C_{\mathrm{Cg}}$ are the liquid and gas phase $\hbox {CO}_2$ concentrations; $m_{\mathrm{H}}$, $m_{\mathrm{C}}$ are the pore averaged concentrations of $\hbox {H}_2\hbox {O}$ and $\hbox {CO}_2$,

6 $$\begin{aligned} m_{\mathrm{H}} = s_{{ l}}C_{\mathrm{W}}+s_{\mathrm{g}}C_{\mathrm{Hg}}, \quad m_{\mathrm{C}} =s_{{ l}}C_{\mathrm{C}{} { l}} + s_{\mathrm{g}}C_{\mathrm{Cg}} ; \end{aligned}$$

and $Q_{{ l}}$, $Q_{\text{ g }}$ are, respectively, the volumetric flow rates of the liquid and gas phases exiting the pore,

7 $$\begin{aligned} Q_{{ l}}= {\Lambda }_{{ l}} f(s_{\mathrm{g}})(p_{{ l}} - p), \quad Q_{\text{g }}= {\Lambda }_{\text{g }}g(s_{\mathrm{g}})(p_{\mathrm{g}} - p). \end{aligned}$$

In (7), $f(s_{\mathrm{g}})$ and $g(s_{\mathrm{g}})$ are the relative permeabilities of the liquid and gas phases respectively. As in Chang and Lindquist^6^, we adopt the models^11^

8 $$\begin{aligned} f(s_{\mathrm{g}})=\left\{ \begin{array}{ll} \frac{1}{2}(1-s_{\mathrm{g}})^2(2+s_{\mathrm{g}}), &{}\quad p_{{ l}}\ge p,\\ 1, &{}\quad p_{{ l}}<p, \end{array} \right. \quad\quad g(s_{\mathrm{g}})=\left\{ \begin{array}{ll} s_{\mathrm{g}}^3, &{}\quad p_{\mathrm{g}}\ge p,\\ 0, &{}\quad p_{\mathrm{g}} < p. \end{array} \right. \end{aligned}$$

Equations (4) and (8) are written to reflect the assumption that the outlet reservoir is sufficiently large that it can be assumed to remain pure water (i.e. any dissolved $\hbox {CO}_2$ in the outlet reservoir mixes to negligible concentration and exiting gas bubbles rise due to buoyancy and cannot flow back into the pore). Therefore, under the backflow condition, $p_{{ l}} < p$, (4) and (8) reflect the fact that pure water flows into the pore from the outlet.

The variables ${\Lambda }_{{ l}}$ and ${\Lambda }_{\text{g }}$ are the channel conductivities of the liquid and gas phase between the pore and the outlet reservoir. Conductivity values are modeled from the Hagen-Poiseuille equation, ${\Lambda }_{{ l}}={\pi a^4}/{8\nu _{{ l}} L}$ and ${\Lambda }_{\text{g }}={\pi a^4}/{8\nu _{\text{g }} L}$. The viscosities, $\nu _{{ l}}$ and $\nu _{\text{g }}$, of liquid and gas phases are given in units of Pa s by Lide and Kehiaian^18^,

$$\begin{aligned}\ln (\nu _{{ l}})& =-10.4349-\dfrac{507.881}{(149.39-T)},\\ \nu _{\rm { g }}& =4.6086\times 10^{-8}T+3.6436\times 10^{-11}T^2 - 9.5765\times 10^{-14}T^3 + 6.1091\times 10^{-17}T^4, \end{aligned}$$

over the temperature range $273.15\,{^\circ }{\mathrm{K}}\le T \le 373.15\,{^\circ }{\mathrm{K}}$.

Equations (5) express the equality (between the liquid and gas phases at equilibrium) of the chemical potentials of $\hbox {H}_2$O and $\hbox {CO}_2$. Inherent in our analysis is the assumption that phase changes occur rapidly compared to fluid transport so that equilibrium conditions are essentially maintained. $\mu _{\beta { l}}^\ominus$ and $\mu _{\beta \mathrm{g}}^\ominus$ are the chemical potentials of the species $\beta = \hbox {H}_2$O or $\hbox {CO}_2$ in the liquid and gas phase at standard conditions. $p_{\mathrm{H}}^*$ and $p_{\mathrm{C}}^*$ are standard pressures which we take to be 1 bar. Equations (5) can be rewritten

9 $$\begin{aligned} C_{\mathrm{Hg}}RT = K_{\mathrm{H}}\dfrac{C_{\mathrm{W}}}{C_{\mathrm{W}} +C_{\mathrm{C}{ l}}}, \quad C_{\mathrm{Cg}}RT = K_{\mathrm{C}}\dfrac{C_{\mathrm{C}{ l}}}{C_{\mathrm{W}}+C_{\mathrm{C}{} { l}}}, \end{aligned}$$

where

$$\begin{aligned} K_{\mathrm{H}}(T)=p_{\mathrm{H}}^*\exp [(\mu _{\mathrm{H}{ l}}^\ominus -\mu _{\mathrm{Hg}}^\ominus )/RT], \qquad K_{\mathrm{C}}(T)=p_{\mathrm{C}}^*\exp [(\mu _{\mathrm{C}{ l}}^\ominus -\mu _{\mathrm{Cg}}^\ominus )/RT]. \end{aligned}$$

Over the range of temperatures of interest, $[273.15\,{^\circ }{\mathrm{K}}, 373.15\,{^\circ }{\mathrm{K}}]$, $K_{\mathrm{H}}(T)$ lies in the range $[1.82864\times 10^{-5},1.94313\times 10^{-5}]$ and the ratio $K_{\mathrm{H}}(T)/K_{\mathrm{C}}(T)$ is in the range $[5.76231\times 10^{-4},4.25324\times 10^{-3}]$.

Assuming the liquid phase is perfectly wetting, the capillary pressure in (3b) is modeled by the Young–Laplace equation,

10 $$\begin{aligned} p_c=\dfrac{2\gamma }{r}, \end{aligned}$$

where $\gamma$ is the surface tension and *r* is the radius of the gas bubble. We assume that the gas phase forms as a single bubble in the pore and therefore estimate $r=s_{\mathrm{g}}^{1/3}\mathrm{R}_{\mathrm{pore}}$. The surface tension, $\gamma$, is evaluated by the Eötvös rule,

$$\begin{aligned} \gamma C_{\mathrm{W}}^{-2/3}=\left\{ \begin{array}{ll} \kappa (T_c-T), &{}\quad T \le T_c, \\ 0, &{}\quad T > T_c, \end{array} \right. \end{aligned}$$

where $T_c =647^\circ K$ is the critical temperature for water and $\kappa =2.1\times 10^{-7}$ JK$^{-1}$mol$^{-2/3}$. We also assume the gas phase is ideal; therefore the total pressure of the gas phase is

11 $$\begin{aligned} p_{\mathrm{g}}=C_{\mathrm{Cg}}RT+C_{\mathrm{Hg}} RT. \end{aligned}$$

### The dynamical system

It will be convenient to divide concentration variables by $C_{\mathrm{W }}$, pressure variables by $C_{\mathrm{W }}RT$, flow rates by *V*, and denote the resultant variables using “over-bar” notation (e.g. ${\overline{C}}_{\mathrm{C}{ l}}=C_{\mathrm{C}{} { l}}/C_{\mathrm{W }}$, ${\overline{m}}_{\mathrm{H}}=m_{\mathrm{H}}/C_{\mathrm{W }}$, ${\overline{p}}=p/C_{\mathrm{W }}RT$, ${\overline{K}}_{\mathrm{C}}=K_{\mathrm{C}}/C_{\mathrm{W }}RT$, ${\overline{Q}}=Q/V$). As a result, all variables become dimensionless except for the volumetric flow rates, ${\overline{Q}}$, ${\overline{Q}}_{{ l}}$, ${\overline{Q}}_{\mathrm{g}}$, which have dimension of time$^{-1}$. The natural time variable (through which to introduce dimensionless flow rates) is the quantity ${\overline{Q}}^{-1}$. However, the flow rate ${\overline{Q}}$ will be a critical variable in our analysis. We therefore retain the variable ${\overline{Q}}$ in our equations and consequently keep an explicit non-dimensional time variable in the dynamical system.

We identify the fundamental variables, *w*, *y*, of the dynamical system as

12 $$\begin{aligned} w={\overline{C}}_{\mathrm{Cg}}, \quad 0\le w \le \dfrac{{\overline{K}}_{\mathrm{C}}{\overline{C}}_{\mathrm{C}}}{1+{\overline{C}}_{\mathrm{C}}}{\mathop {=}\limits ^{\mathrm{def}}}\, {\overline{C}}_{\mathrm{Cg}}({\overline{C}}_{\mathrm{C}}); \qquad y=s_{\mathrm{g}}, \quad 0\le y\le 1. \end{aligned}$$

Since the inlet solution is an aqueous phase, we require ${\overline{C}}_{\mathrm{C}} < 1$. Therefore $w \le {\overline{C}}_{\mathrm{Cg}}({\overline{C}}_{\mathrm{C}}) < {\overline{K}}_{\mathrm{C}}$. From (9), the concentrations ${\overline{C}}_{\mathrm{Hg}}$ and ${\overline{C}}_{\mathrm{C}{} { l}}$ can be expressed in terms of *w*:

13 $$\begin{aligned} &{\overline{C}}_{\mathrm{Hg}}={\overline{K}}_{\mathrm{H}} \dfrac{{\overline{K}}_{\mathrm{C}}-w}{{\overline{K}}_{\mathrm{C}}}, \quad\quad {\overline{C}}_{\mathrm{Hg}} \in \left[ \dfrac{{\overline{K}}_{\mathrm{H}}}{1+{\overline{C}}_{\mathrm{C}}}, {\overline{K}}_{\mathrm{H}}\right] ; \\  &{\overline{C}}_{\mathrm{C}{ l}}=\dfrac{w}{{\overline{K}}_{\mathrm{C}}-w}, \quad\quad\quad {\overline{C}}_{\mathrm{C}{} { l}} \in [0, {\overline{C}}_{\mathrm{C}}]. \end{aligned}$$

Note that the definition of ${\overline{C}}_{\mathrm{Cg}}({\overline{C}}_{\mathrm{C}})$ in (12) and the relation for ${\overline{C}}_{\mathrm{C}{} { l}}$ in (13) gives the identity ${\overline{C}}_{\mathrm{C}}={\overline{C}}_{\mathrm{Cg}}({\overline{C}}_{\mathrm{C}})/({\overline{K}}_{\mathrm{C}}-{\overline{C}}_{\mathrm{Cg}}({\overline{C}}_{\mathrm{C}}))={\overline{C}}_{\mathrm{C}{ l}}({\overline{C}}_{\mathrm{Cg}}({\overline{C}}_{\mathrm{C}}))$.

From (10), (11) and (3b), the dimensionless pore phase pressures are expressed in terms of *w* and *y* as

14 $$\begin{aligned}&{\overline{p}}_c={\overline{s}}y^{-1/3}, \quad {\overline{p}}_{\mathrm{g}}=\left( 1-\dfrac{{\overline{K}}_{\text{ H }}}{{\overline{K}}_{\mathrm{C}}}\right) w+{\overline{K}}_{\text{ H }},\\ &{\overline{p}}_{{ l}}=\left( 1-\dfrac{{\overline{K}}_{\mathrm{H}}}{{\overline{K}}_{\text{ C }}}\right) w+{\overline{K}}_{\mathrm{H}} -{\overline{s}}y^{-1/3},\end{aligned}$$

where we have defined ${\overline{s}}=2\gamma /(\mathrm{R}_{\mathrm{pore}}C_{\mathrm{W}}RT)$. From (14) we note ${\overline{p}}_{{ l}}< {\overline{p}}_{\mathrm{g}}$. Equations (4) become

15a $$\begin{aligned} \dfrac{d{\overline{m}}_{\mathrm{H}}}{dt}&=\left\{ \begin{array}{ll} {\overline{Q}}-{\overline{Q}}_{{ l}}(w,y)- {\overline{C}}_{\mathrm{Hg}}(w){\overline{Q}}_{\mathrm{g}}(w,y), &{}\quad {\overline{p}}_{\mathrm{g}}>{\overline{p}}, \\ {\overline{Q}}-{\overline{Q}}_{{ l}}(w,y), &{}\quad {\overline{p}}\ge {\overline{p}}_{\mathrm{g}}, \end{array}\right\} \nonumber \\&{\mathop {=}\limits ^{\mathrm{def}}}{\Delta }_{\mathrm{H}}(w,y), \end{aligned}$$

15b $$\begin{aligned} \dfrac{d{\overline{m}}_{\mathrm{C}}}{dt}&=\left\{ \begin{array}{lll} {\overline{C}}_{\mathrm{C}}{\overline{Q}}-{\overline{C}}_{\mathrm{C}{ l}}(w){\overline{Q}}_{{ l}}(w,y)-w{\overline{Q}}_{\mathrm{g}}(w,y), &{}\quad {\overline{p}}_{\mathrm{g}}>{\overline{p}}_{{ l}}>{\overline{p}}, \\ {\overline{C}}_{\mathrm{C}}{\overline{Q}}-w{\overline{Q}}_{\mathrm{g}}(w,y), &\quad {}{\overline{p}}_{\mathrm{g}}>{\overline{p}}\ge {\overline{p}}_{{ l}},\\ {\overline{C}}_{\mathrm{C}}{\overline{Q}}, &{}\quad {\overline{p}}\ge {\overline{p}}_{\mathrm{g}}>{\overline{p}}_{{ l}}, \end{array} \right\} \nonumber \\&\quad {\mathop {=}\limits ^{\mathrm{def}}} \mathrm{\Delta }_{\mathrm{C}}(w,y). \end{aligned}$$

We can obtain alternate expressions to (15) for ${\Delta }_{\mathrm{H}}$ and $\mathrm{\Delta }_{\mathrm{C}}$. Starting from (6), we have

16 $$\begin{aligned} {\overline{m}}_{\mathrm{H}}(w,y)=1-y+y{\overline{C}}_{\text{ Hg }}(w), \quad {\overline{m}}_{\mathrm{C}}(w,y)=(1-y){\overline{C}}_{\mathrm{C}{ l}}(w)+yw.\end{aligned}$$

Taking the time derivative of (16) gives the alternate expressions,

17a $$\begin{aligned} \mathrm{\Delta }_{\mathrm{H}}(w,y)&=y\dfrac{d{\overline{C}}_{\mathrm{Hg}}(w)}{dw}\dfrac{dw}{dt}+({\overline{C}}_{\mathrm{Hg}}(w)-1)\dfrac{dy}{dt}\nonumber \\&{\mathop {=}\limits ^{\mathrm{def}}}G_1 (w,y)\dfrac{dw}{dt}+G_2(w)\dfrac{dy}{dt}, \end{aligned}$$

17b $$\begin{aligned} \mathrm{\Delta }_{\mathrm{C}}(w,y)&=\left[ (1-y)\dfrac{d{\overline{C}}_{\mathrm{C}{} { l}}(w)}{dw}+y\right] \dfrac{dw}{dt}+(w-{\overline{C}}_{\mathrm{C}{} { l}}(w))\dfrac{dy}{dt}\nonumber \\&{\mathop {=}\limits ^{\mathrm{def}}}F_1 (w,y)\dfrac{dw}{dt}+F_2(w)\dfrac{dy}{dt}, \end{aligned}$$

where for notational brevity, we have introduced the functions $G_1$, $G_2$, $F_1$, $F_2$. Solving (17) for *dw*/*dt* and *dy*/*dt* gives the dynamical system

18 $$\begin{aligned} \begin{array}{l} \dfrac{dw}{dt}=\dfrac{\mathrm{\Delta }_{\mathrm{C}}(w,y)G_2(w)-F_2(w)\mathrm{\Delta }_{\mathrm{H}}(w,y)}{F_1 (w,y)G_2(w)-F_2(w)G_1(w,y)},\\ \\ \dfrac{dy}{dt}=\dfrac{F_1(w,y)\mathrm{\Delta }_{\mathrm{H}}(w,y)-\mathrm{\Delta }_{\mathrm{C}}(w,y)G_1(w,y)}{F_1 (w,y)G_2(w)-F_2(w)G_1(w,y)}. \end{array} \end{aligned}$$

Using (13) to evaluate the concentrations and concentration derivatives in (17) and noting that ${\overline{K}}_{\mathrm{H}}<2\times 10^{-5}$ and ${\overline{K}}_{\mathrm{H}}/{\overline{K}}_{\mathrm{C}}<O(10^{-3})$ over the range of temperatures of interest, we have, to good approximation,

19 $$\begin{aligned} {\overline{C}}_{\mathrm{Hg}}(w)\approx 0 \Rightarrow G_2(w)\approx -1; \qquad \dfrac{d{\overline{C}}_{\mathrm{Hg}}(w)}{dw}=-\dfrac{{\overline{K}}_{\mathrm{H}}}{{\overline{K}}_{\mathrm{C}}}\approx 0 \Rightarrow G_1(w,y)\approx 0. \end{aligned}$$

The statement ${\overline{K}}_{\mathrm{H}}/{\overline{K}}_{\mathrm{C}}\ll 1$ has a physical consequence, namely that the $\hbox {H}_2$O component in the gas phase remains small in comparison to the $\hbox {CO}_2$ component. Approximations (19) imply the simplifications: of (14) to

14′ $$\begin{aligned} {\overline{p}}_c(y)={\overline{s}}y^{-1/3},\qquad {\overline{p}}_{\mathrm{g}}(w)\approx w,\qquad {\overline{p}}_{{ l}}(w,y)\approx w-{\overline{s}}y^{-1/3}; \end{aligned}$$

of (15) to

15′a $$\begin{aligned}&{\Delta }_{\mathrm{H}}(w,y) \approx {\overline{Q}}-{\overline{Q}}_{{ l}}(w,y), \end{aligned}$$

15′b $$\begin{aligned}&{\Delta }_{\mathrm{C}}(w,y)=\left\{ \begin{array}{lll} {\overline{C}}_{\mathrm{C}}{\overline{Q}}-{\overline{C}}_{\mathrm{C}{ l}}(w){\overline{Q}}_{{ l}}(w,y)-w{\overline{Q}}_{\mathrm{g}}(w,y), &{}\quad {\overline{p}}_{\mathrm{g}}(w)>{\overline{p}}_{{ l}}(w,y)>{\overline{p}}, \\ {\overline{C}}_{\mathrm{C}}{\overline{Q}}-w{\overline{Q}}_{\text{ g }}(w,y), &{}\quad {\overline{p}}_{\mathrm{g}}(w)>{\overline{p}} \ge {\overline{p}}_{{ l}}(w,y),\\ {\overline{C}}_{\mathrm{C}}{\overline{Q}}, &{}\quad {\overline{p}}\ge {\overline{p}}_{\mathrm{g}}(w)>{\overline{p}}_{{ l}}(w,y); \end{array} \right. \qquad \end{aligned}$$

and of (18) to

18′ $$\begin{aligned} \dfrac{dw}{dt}\approx \dfrac{F_2(w)\mathrm{\Delta }_{\mathrm{H}}(w,y)+\mathrm{\Delta }_{\mathrm{C}}(w,y)}{F_1 (w,y)}{\mathop {=}\limits ^{\mathrm{def}}}F(w,y),\quad \dfrac{dy}{dt}\approx -\mathrm{\Delta }_{\mathrm{H}}(w,y){\mathop {=}\limits ^{\mathrm{def}}}G(w,y). \end{aligned}$$

The second and third equations of (14′) hold only for $w\gg {\overline{K}}_{\mathrm{H}}=O(10^{-5})$. This approximation will ultimately effect computation of phase space trajectories for values of $w \lesssim 10^{-4}$. Our results will demonstrate that the region of phase space governing bubble formation occurs well away from “small *w*” values, and we proceed with the approximation (14′).

In (18′) we have used a standard notation, *F*(*w*, *y*) and *G*(*w*, *y*), for the right-hand side of this two-by-two system of ODEs. The dynamical system we consider is (18′) with: $\mathrm{\Delta }_{\mathrm{H}}$, ${\Delta }_{\mathrm{C}}$ defined by (15$^\prime$); $F_1$, $F_2$ defined by (17b); ${\overline{Q}}_{{ l}}$, ${\overline{Q}}_{\mathrm{g}}$ given by (7) and (8) appropriately normalized,

7′a $$\begin{aligned} {\overline{Q}}_{{ l}}(w,y)= \left\{ \begin{array}{lll} \overline{{\Lambda }}_{{ l}}f(y)({\overline{p}}_{{ l}}(w,y)-{\overline{p}}), &{}\quad {\overline{p}}_{{ l}}(w,y)>{\overline{p}},\\ 0, &{}\quad {\overline{p}}_{{ l}}(w,y)={\overline{p}},\\ \overline{{\Lambda }}_{{ l}}({\overline{p}}_{{ l}}(w,y)-{\overline{p}}), &{}\quad {\overline{p}}_{{ l}}(w,y)<{\overline{p}}, \end{array}\right. \\ \end{aligned}$$

7′b $$\begin{aligned} {\overline{Q}}_{\mathrm{g}}(w,y)=\left\{ \begin{array}{ll} \overline{{\Lambda }}_{\mathrm{g}}g(y)({\overline{p}}_{\mathrm{g}}(w) -{\overline{p}}), &{}\quad {\overline{p}}_{\mathrm{g}}(w)>{\overline{p}},\\ 0, &{}\quad {\overline{p}}_{\mathrm{g}}(w)\le {\overline{p}}, \end{array} \right. \end{aligned}$$

where $\overline{{\Lambda }}_\alpha = {\Lambda }_\alpha C_{\mathrm{W}}RT/V$, $\alpha =l, \text{ g }$; and the phase pressures given by (14′).

## Analysis of the dynamical system

### The curves ${{\overline{Q}}_{{ l}}=0}$ and ${{\overline{Q}}_{\rm g}=0}$

The physical *w*, *y* phase space for (18′) is $0 \le w \le {\overline{C}}_{\mathrm{Cg}}({\overline{C}}_{\mathrm{C}})$, $0 < y \le 1$. It contains two important curves. The first is the curve ${\Gamma }_{l\mathrm{0}}$ defined by the condition ${\overline{Q}}_{{ l}}(w,y)={\overline{p}}_{{ l}}(w,y)-{\overline{p}}=0$ which, from (14′), can be written either as

20 $$\begin{aligned} y_{\tiny{ l0}}(w)=({\overline{s}}/(w-{\overline{p}}))^3 \qquad \text{ or } \qquad w_{\tiny{ l0}}(y)={\overline{p}}+{\overline{s}}/y^{1/3}. \end{aligned}$$

From (20), $w_{\tiny { l0}}(1)={\overline{p}}+{\overline{s}}$. Note that, for any point $(w,y)\in [0, {\overline{C}}_{\mathrm{Cg}}({\overline{C}}_{\mathrm{C}})]\times (0,1]$ on ${\Gamma }_{l\mathrm{0}}$, we have ${\overline{C}}_{\mathrm{Cg}}({\overline{C}}_{\mathrm{C}}) \ge {\overline{p}}_{\mathrm{g}}={\overline{p}}+{\overline{s}}y^{-1/3}>{\overline{p}}+{\overline{s}}$, by which we confirm that the value ${\overline{p}}+{\overline{s}}$ lies strictly in the range $0< {\overline{p}}+{\overline{s}} < {\overline{C}}_{\mathrm{Cg}}({\overline{C}}_{\mathrm{C}})$. Thus, $0 < y_{\tiny { l0}}(w) \le 1$ for all $w \in [{\overline{p}}+{\overline{s}}, {\overline{C}}_{\mathrm{Cg}}({\overline{C}}_{\mathrm{C}})]$. Furthermore, from (20) we note that the slope $dy_{\tiny { l0}}(w)/dw < 0$ for $w\in [{\overline{p}}+{\overline{s}}, {\overline{C}}_{\mathrm{Cg}}({\overline{C}}_{\mathrm{C}})]$. Thus $y_{\tiny { l0}}(w)$ decreases from its maximum value $y_{\tiny { l0}}({\overline{p}}+{\overline{s}}) = 1$ over the domain $[{\overline{p}}+{\overline{s}}, {\overline{C}}_{\mathrm{Cg}}({\overline{C}}_{\mathrm{C}})]$. At the $w = {\overline{C}}_{\mathrm{Cg}}({\overline{C}}_{\mathrm{C}})$ endpoint of ${\Gamma }_{l\mathrm{0}}$ we have

$$\begin{aligned} 1> y_{\tiny { l0}}({\overline{C}}_{\mathrm{Cg}}({\overline{C}}_{\mathrm{C}})) = \left( \dfrac{{\overline{s}}}{{\overline{C}}_{\mathrm{Cg}} ({\overline{C}}_{\mathrm{C}}) - {\overline{p}}}\right) ^3 > 0. \end{aligned}$$

The second important curve in the phase space, ${\Gamma }_{\mathrm{g{0}}}$, is defined by

21 $$\begin{aligned} {\overline{Q}}_{\text{ g }} = {\overline{p}}_{\mathrm{g}}(w) - {\overline{p}} = w -{\overline{p}} = 0; \end{aligned}$$

i.e., ${\Gamma }_{\mathrm{g{0}}}$ is the vertical line $w={\overline{p}}$.

The curves ${\Gamma }_{l \mathrm{0}}$ and ${\Gamma }_{\mathrm{g}{0}}$ divide the phase space into three regions. Let: ${\Gamma }_{++}$ denote the region of the phase space where ${\overline{p}}_{\text{ g }}(w)> {\overline{p}}_{{ l}}(w,y) > {\overline{p}}$; ${\Gamma }_{-+}$ denote the region where ${\overline{p}}_{\text{ g }}(w)> {\overline{p}} > {\overline{p}}_{{l}}(w,y)$; and ${\Gamma }_{--}$ denote the region where ${\overline{p}}> {\overline{p}}_{\text{ g }}(w) > {\overline{p}}_{{l}}(w,y)$. Figure 2 provides sketches of ${\Gamma }_{{{ l}0}}$, ${\Gamma }_{\mathrm{g{0}}}$ and indicates the regions ${\Gamma }_{++}$, ${\Gamma }_{-+}$ and ${\Gamma }_{--}$.

### Direction fields

As the dynamical system (18′) is complex and the details of its analysis are not straightforward, to respect article length we have relegated most of these details to a series of appendices in the accompanying supplementary material. The manuscript text references the appropriate appendix as needed.

#### The direction field ${dy/dt=G(w,y)}$

The direction field *dy*/*dt* is analyzed in Appendix A of the supplementary material by considering the isoclines of *G*(*w*, *y*). All isoclines in the region ${\Gamma }_{--}\cup {\Gamma }_{\mathrm{g{0}}}\cup {\Gamma }_{-+}\cup {\Gamma }_{l\mathrm{0}}$ are of the monotonic form (A.9) given in Appendix A and are negative-valued. The curve ${\Gamma }_{\mathrm{g{0}}}$ is not an isocline; explicit evaluation using (18′), (15′a), (7′a), (3b), (21) and (14′) gives $G({\overline{p}},y) = -\overline{{\Lambda }}_{{ l}}{\overline{s}}y^{-1/3} - {\overline{Q}} < 0$. From (18′) and (15′a) it is straightforward to see that ${\Gamma }_{l\mathrm{0}}$ is the $G(w,y)=-{\overline{Q}}$ isocline.

The *G*(*w*, *y*) isoclines in ${\Gamma }_{++}$ have the form (A.4) of Appendix A and are “concave-up” in the direction $w \rightarrow \infty$ (see Fig. A.1(a) of Appendix A). The only region where *G*(*w*, *y*) can take positive values (implying gas bubble growth) is ${\Gamma }_{++}$.

**Figure 3:**
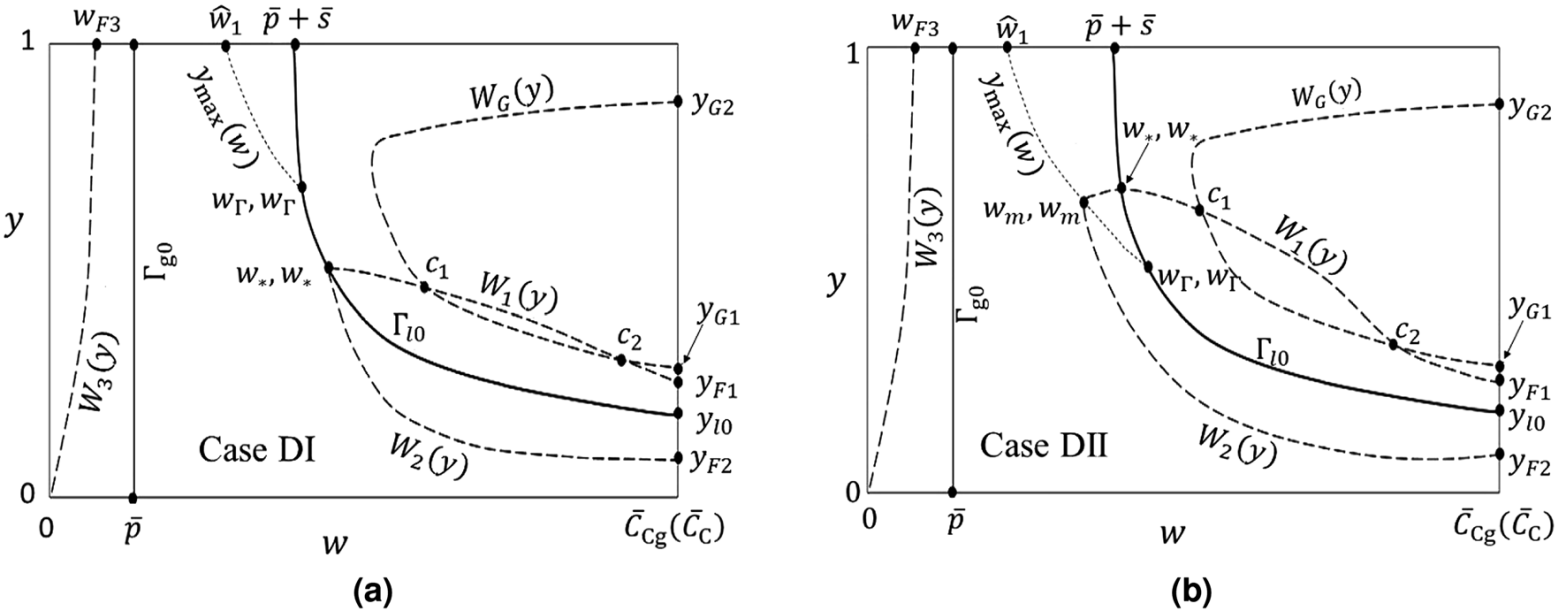
Sketch of the relevant phase space curves and regions discussed in the text and Appendices A to E in the supplementary material. The difference in the curve $W_2(y)$ in cases DI and DII is discussed in Appendix D.

There exists a maximum value of ${\overline{Q}}$ such that, for flow strengths above this value, the isocline $G(w,y)=0$ does not (and hence no positive-valued isoclines can) appear in the physical phase space and no gas bubble growth is possible. Computation for this maximum flow strength ${\overline{Q}}_{\mathrm{max}}=\alpha _{\mathrm{max}}\overline{{\Lambda }}_l{\overline{s}}$, is illustrated in Fig. A.1(b) of Appendix A. With the condition ${\overline{Q}}<{\overline{Q}}_{\mathrm{max}}$ satisfied, the form of the isocline $G(w,y)=0$ is shown in Fig. 3 where, following the notation introduced in Appendix A, it is referred to as $W_{{ G}}(y)$. $W_{{ G}}(y)$ intersects the phase space boundary $w = {\overline{C}}_{\mathrm{Cg}}({\overline{C}}_{\mathrm{C}})$ at the values $y_{{ G1}}$ and $y_{{ G2}}$.

#### The direction field ${dw/dt=F(w,y)}$

The direction field *F*(*w*, *y*) is more complex and we have relied on estimation procedures to determine its behavior. To proceed, we express *F*(*w*, *y*) in (18′) as

22 $$\begin{aligned} F(w,y)=\dfrac{({\overline{K}}_{\mathrm{C}}-w)[F_2(w)\mathrm{\Delta }_{\mathrm{H}}(w,y)+\mathrm{\Delta }_{\mathrm{C}}(w,y)]}{({\overline{K}}_{\mathrm{C}}-w)F_1(w,y)} {\mathop {=}\limits ^{\mathrm{def}}} \dfrac{{\widetilde{F}}(w,y)}{({\overline{K}}_{\mathrm{C}}-w)F_1(w,y)}. \end{aligned}$$

Since the denominator in (22) is always positive, we note that *F* and ${\widetilde{F}}$ have the same zero-points and signs. Using (15$^\prime$), and (7$^\prime$) we obtain the following expressions for ${\widetilde{F}}$:

23a $$\begin{aligned} {\widetilde{F}}(w,y)|_{{\Gamma }_{++}}&=({\overline{K}}_{\mathrm{C}}-w)\{(F_2(w)+{\overline{C}}_{\mathrm{C}}){\overline{Q}} - w[\overline{{\Lambda }}_{{ l}}f(y)+\overline{{\Lambda }}_{\mathrm{g}}g(y)](w-{\overline{p}})\nonumber \\&\quad + w\overline{{\Lambda }}_{{ l}}f(y){\overline{s}}y^{-1/3}\}; \end{aligned}$$

23b $$\begin{aligned} {\widetilde{F}}(w,y)|_{{\Gamma }_{l\mathrm{0}}}&=({\overline{K}}_{\mathrm{C}} - w)\{(F_2(w) + {\overline{C}}_{\mathrm{C}}){\overline{Q}} - w\overline{{\Lambda }}_{\mathrm{g}}{\overline{s}}y_{{\tiny l0}}^{8/3} \}; \end{aligned}$$

23c $$\begin{aligned} {\widetilde{F}}(w,y)|_{{\Gamma }_{-+}}&=({\overline{K}}_{\mathrm{C}}-w)\{(F_2(w) +{\overline{C}}_{\mathrm{C}}){\overline{Q}} - [F_2(w)\overline{{\Lambda }}_{{ l}} + w\overline{{\Lambda }}_{\mathrm{g}}y^3](w-{\overline{p}})\nonumber \\&\quad + F_2(w)\overline{{\Lambda }}_{{ l}}{\overline{s}}y^{-1/3}\}; \end{aligned}$$

23d $$\begin{aligned} {\widetilde{F}}(w, y)|_{{\Gamma }_{\mathrm{g{0}}}}&=({\overline{K}}_{\mathrm{C}}-{\overline{p}})\{(F_2({\overline{p}}) +{\overline{C}}_{\mathrm{C}}){\overline{Q}} + F_2({\overline{p}})\overline{{\Lambda }}_{{ l}}{\overline{s}}y^{-1/3}\}; \end{aligned}$$

23e $$\begin{aligned} {\widetilde{F}}(w,y)|_{{\Gamma }_{--}}&=({\overline{K}}_{\mathrm{C}}-w)\{(F_2(w) +{\overline{C}}_{\mathrm{C}}){\overline{Q}} - F_2(w)\overline{{\Lambda }}_{{ l}}(w-{\overline{p}})\nonumber \\&\quad + F_2(w)\overline{{\Lambda }}_{{ l}}{\overline{s}}y^{-1/3}\}; \end{aligned}$$

where by (17b) and (13), $F_2(w)=w-w/({\overline{K}}_{\mathrm{C}}-w)$. Note $F_2(w) < 0$ providing ${\overline{K}}_{\mathrm{C}} < 1$, which is indeed the case in our numerical computations (see Table 1). However, $F_2(w) + {\overline{C}}_{\mathrm{C}} > 0$ in the domain of interest. The expressions for ${\widetilde{F}}(w,y)$ in (23) are continuous across the curves ${\Gamma }_{{{ l}0}}$ and ${\Gamma }_{\mathrm{g0}}$.

We introduce four restrictions on the range of ${\overline{Q}}$ to make our estimations tractable. Evaluating (23b) at the point $({\overline{p}} + {\overline{s}}, 1)$, we have

$$\begin{aligned} {\widetilde{F}}({\overline{p}} + {\overline{s}},1)=({\overline{K}}_{\mathrm{C}}-{\overline{p}} - {\overline{s}})\left[ F_2({\overline{p}} + {\overline{s}}) + {\overline{C}}_{\mathrm{C}}\right] \left\{ {\overline{Q}}-\dfrac{({\overline{p}} + {\overline{s}})\overline{{\Lambda }}_{\mathrm{g}}{\overline{s}}}{\left[ F_2({\overline{p}} + {\overline{s}}) + {\overline{C}}_{\mathrm{C}}\right] }\right\} . \end{aligned}$$

Restricting ${\overline{Q}} < {({\overline{p}} + {\overline{s}})\overline{{\Lambda }}_{\mathrm{g}}{\overline{s}}}/{\left[ F_2({\overline{p}} + {\overline{s}}) + {\overline{C}}_{\mathrm{C}}\right] }$ will guarantee that ${\widetilde{F}}({\overline{p}} + {\overline{s}},1) < 0$. Evaluating (23b) at the point $({\overline{C}}_{\mathrm{Cg}}({\overline{C}}_{\mathrm{C}}), y_{\tiny { l0}}({\overline{C}}_{\mathrm{Cg}}({\overline{C}}_{\mathrm{C}})))$, we have

$$\begin{aligned} {\widetilde{F}}({\overline{C}}_{\mathrm{Cg}}({\overline{C}}_{\text{ C }}),y_{\tiny { l0}}({\overline{C}}_{\mathrm{Cg}}({\overline{C}}_{\mathrm{C}}))) = ({\overline{K}}_{\mathrm{C}}-{\overline{C}}_{\mathrm{Cg}} ({\overline{C}}_{\mathrm{C}})){\overline{C}}_{\mathrm{Cg}}({\overline{C}}_{\mathrm{C}})\left\{ {\overline{Q}}- \overline{{\Lambda }}_{\mathrm{g}}{\overline{s}}\left( \dfrac{{\overline{s}}}{{\overline{C}}_{\mathrm{Cg}}({\overline{C}}_{\mathrm{C}}) -{\overline{p}}}\right) ^{8}\right\} . \end{aligned}$$

Restricting ${\overline{Q}} > \overline{{\Lambda }}_{\mathrm{g}}{\overline{s}}\left[ {\overline{s}}/({\overline{C}}_{\mathrm{Cg}}({\overline{C}}_{\mathrm{C}})-{\overline{p}})\right] ^8$ will guarantee that ${\widetilde{F}}({\overline{C}}_{\mathrm{Cg}}({\overline{C}}_{\mathrm{C}}),y_{\tiny { l0}}({\overline{C}}_{\mathrm{Cg}}({\overline{C}}_{\mathrm{C}}))) > 0$. Evaluating (23d) at the point $({\overline{p}}, 1)$ we have

$$\begin{aligned} {\widetilde{F}}({\overline{p}},1)=({\overline{K}}_{\mathrm{C}}- {\overline{p}})\left\{ (F_2 ({\overline{p}})+{\overline{C}}_{\mathrm{C}}){\overline{Q}} +F_2({\overline{p}})\overline{{\Lambda }}_l{\overline{s}}\right\} . \end{aligned}$$

Restricting ${\overline{Q}}<-F_2({\overline{p}})\overline{{\Lambda }}_l {\overline{s}}/(F_2({\overline{p}})+{\overline{C}}_{\mathrm{C}})$ will guarantee that ${\widetilde{F}}({\overline{p}}, 1) < 0$. We also note from the previous section that the restriction ${\overline{Q}}<{\overline{Q}}_{\mathrm{max}}$ guarantees that there is a region $G(w,y)>0$ within ${\Gamma }_{++}$. We combine these restrictions on ${\overline{Q}}$ into the single statement,

24 $$\begin{aligned} \left( \dfrac{{\overline{s}}}{{\overline{C}}_{\mathrm{Cg}} ({\overline{C}}_{\mathrm{C}})-{\overline{p}}}\right) ^8< \dfrac{{\overline{Q}}}{\overline{{\Lambda }}_{\mathrm{g}}{\overline{s}}} < \min \left( \dfrac{{\overline{p}}+{\overline{s}}}{F_2({\overline{p}}+{\overline{s}}) +{\overline{C}}_{\mathrm{C}}}, \dfrac{-\overline{{\Lambda }}_{{ l}}F_2({\overline{p}})}{\overline{{\Lambda }}_{\mathrm{g}}(F_2({\overline{p}}) +{\overline{C}}_{\mathrm{C}})}, \alpha _{\mathrm{max}}\dfrac{\overline{{\Lambda }}_{{ l}}}{\overline{{\Lambda }}_{\mathrm{g}}}\right) . \end{aligned}$$

The behavior of ${\widetilde{F}}(w,y)$ in the phase space is discussed next.

##### ${\widetilde{{{F}}}{(w,y)}}$ on ${{\Gamma }_{{{l0}}}}$

In Appendix B (supplementary material) we show ${\widetilde{F}}(w,y)$ has the following behavior on ${\Gamma }_{l\mathrm{0}}$:

B.9 $$\begin{aligned} {\widetilde{F}}(w,y)|_{{\Gamma }_{l\mathrm{0}}}\left\{ \begin{array}{lll} < 0,\qquad w\in [{\overline{p}}+{\overline{s}}, w_*),\\ = 0,\qquad w=w_*, \\ > 0,\qquad w\in (w_*,{\overline{C}}_{\mathrm{Cg}}({\overline{C}}_{\mathrm{C}})]. \end{array} \right. \end{aligned}$$

The existence of the point $(w_*,y_*=y_{{\tiny l0}}(w_*))$ within the physical domain is guaranteed by assumption (24). The point $(w_*,y_*)$ is illustrated in Fig. 3.

##### ${\widetilde{{{F}}}{(w,y)}}$ on ${{\Gamma }}_{{\mathrm{g0}}}$

Using (23d) we see that the derivative $\partial {\widetilde{F}}({\overline{p}},y))/\partial y = -F_2({\overline{p}})({\overline{K}}_{\mathrm{C}}-{\overline{p}}) \overline{{\Lambda }}_l{\overline{s}}y^{-4/3}/3 > 0$. Hence ${\widetilde{F}}({\overline{p}},y)$ is a strictly increasing function of *y*. From restriction (24) we have ${\widetilde{F}}({\overline{p}},1) < 0$. Therefore, ${\widetilde{F}}({\overline{p}},y)<0$ for all $y\in (0,1]$.

##### ${\widetilde{{{F}}}{(w,y)}}$ in ${{\Gamma }_{++}}$

**Figure 4:**
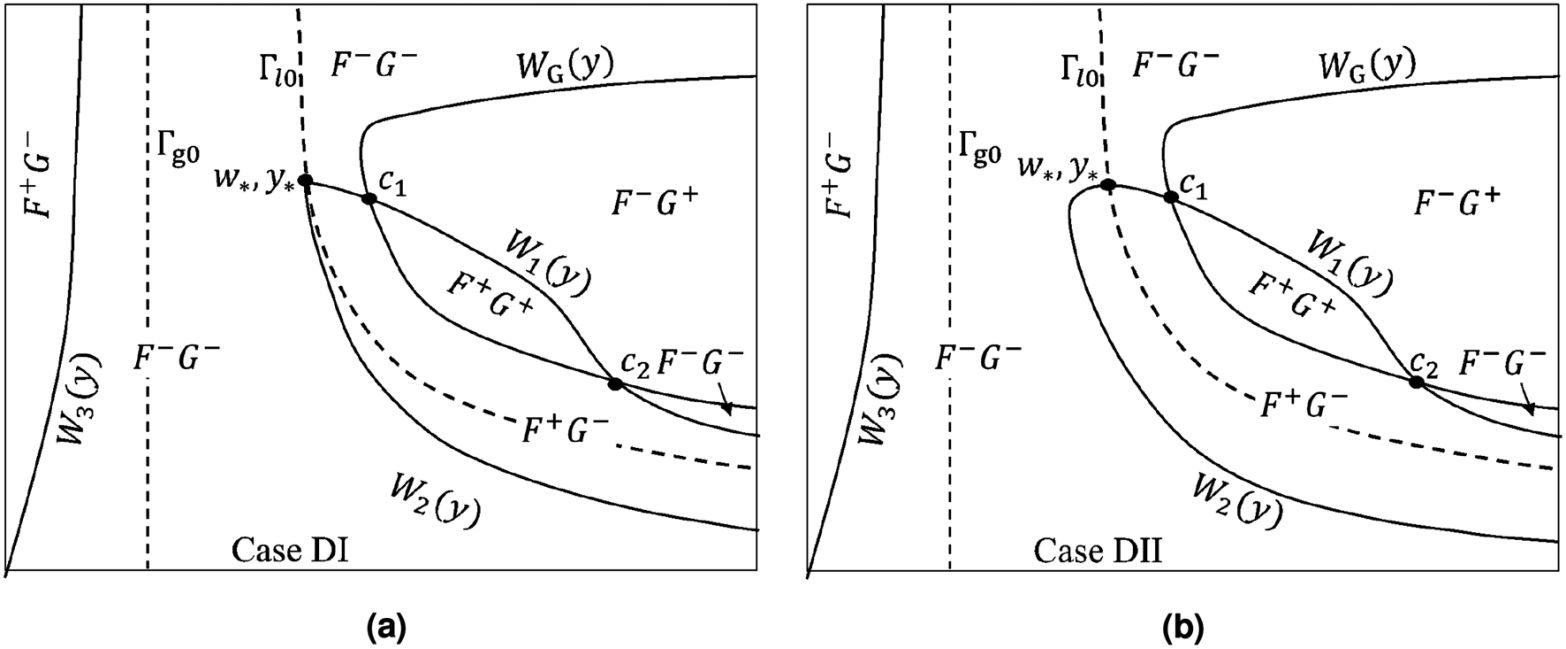
Signs for the direction fields *F*(*w*, *y*), *G*(*w*, *y*) in the phase space for (**a**) Case DI and (**b**) Case DII. $F^+G^-$ denotes a region where $F(w,y)>0$, $G(w,y)<0$, etc.

${\widetilde{F}}(w,y)|_{{\Gamma }_{++}}$ is analyzed in Appendix C (supplementary material). There we prove the existence of a single curve segment, $W_1(y)$, in ${\Gamma }_{++}$ on which ${\widetilde{F}}(w,y)|_{{\Gamma }_{++}}=0$. $W_1(y)$ connects the point $(w_*, y_*)$ on ${\Gamma }_{l\mathrm{0}}$ to a point $({\overline{C}}_{\mathrm{Cg}}({\overline{C}}_{\mathrm{C}}), y_{\tiny \text{ F1 }})$ where $y_{\tiny \text{ F1 }}$ lies in the interval $(y_{\tiny l0}({\overline{C}}_{\mathrm{Cg}}({\overline{C}}_{\mathrm{C}})), y_{\mathrm{G1}})$. By Lemma C of Appendix C, $y_{\text{ F1 }} < y_*$. $W_1(y)$ is a continuous function of *y*; for each value of $y\in [y_{\text{ F1 }}, y_*]$ there is a unique value $W_1(y)$. A sketch of this curve segment is shown in Fig. 3. Regions where ${\widetilde{F}}(w,y)|_{{\Gamma }_{++}}\gtrless 0$ are indicated in Fig. 4.

##### ${\widetilde{{{F}}}{(w,y)}}$ in ${{\Gamma }_{-+}}$

${\widetilde{F}}(w,y)|_{{\Gamma }_{-+}}$ is analyzed in Appendix D (supplementary material). There we show ${\Gamma }_{-+}$ contains a curve, $y_{\mathrm{max}}(w)$, on which $\partial {\widetilde{F}}(w,y)/\partial y|_{{\Gamma }_{-+}}=0$. $y_{\mathrm{max}}(w)$ joins a unique point $({\widehat{w}}_1,1)$, where ${\overline{p}}< {\widehat{w}}_1 < {\overline{p}}+{\overline{s}}$, to a unique point $(w_{{\Gamma }},y_{{\Gamma }})$ on ${\Gamma }_{l\mathrm{0}}$. In addition, ${\widetilde{F}}(w,y)|_{{\Gamma }_{-+}}=0$ in ${\Gamma }_{-+}$ on a single curve segment $W_2(y)$ joining the point $(w_*,y_*)$ to $({\overline{C}}_{\mathrm{Cg}}({\overline{C}}_{\mathrm{C}}), y_{\tiny \text{ F2 }})$, where $y_{\text{ F2 }}$ is a unique value satisfying $0<y_{\text{ F2 }} < y_{\tiny l0}({\overline{C}}_{\mathrm{Cg}}({\overline{C}}_{\mathrm{C}}))$. The curve $W_2(y)$ can have two possible forms, depending on the relative size of $w_{{\Gamma }}$ and $w_*$ (cases DI and DII in Appendix D). The curves $y_{\mathrm{max}}(w)$ and $W_2(y)$ are sketched in Fig. 3 and the points $({\widehat{w}}_1, 1)$, $(w_{\Gamma }, y_{\Gamma })$ and $({\overline{C}}_{\mathrm{Cg}}({\overline{C}}_{\mathrm{C}}), y_{\tiny \text{ F2 }})$ are indicated. Regions where ${\widetilde{F}}(w,y)|_{{\Gamma }_{-+}}\gtrless 0$ are shown in Fig. 4.

##### ${\widetilde{{{F}}}{(w,y)}}$ in ${{\Gamma }_{--}}$

The function ${\widetilde{F}}(w,y)|_{{\Gamma }_{--}}$ is analyzed in Appendix E (supplementary material). On the boundary $y = 1$ there exists a unique value $w_{F3}\in (0,{\overline{p}})$ such that ${\widetilde{F}}(w_{F3},1) = 0$. In the interior of ${\Gamma }_{--}$, ${\widetilde{F}}(w,y)|_{{\Gamma }_{--}} = 0$ only on a curve $W_3(y)$ having the properties: $\lim _{y\rightarrow 0^+} W_3(y)\rightarrow 0$, $W_3(1) = w_{F3}$, and $dW_3(y)/dy > 0$. Fig. 3 shows a sketch of the curve $W_3(y)$ and the point $(w_{F3},1)$. Regions where ${\widetilde{F}}(w,y)|_{{\Gamma }_{--}}\gtrless 0$ are indicated in Fig.  4.

### Critical point existence

A point $(w_c,y_c)$ is a critical point of (18′) if $F(w_c,y_c)=G(w_c,y_c)=0$. From (18′), we conclude $(w_c,y_c)$ is a critical point of (18′) iff $\mathrm{\Delta }_{\mathrm{H}}(w_c,y_c) = \mathrm{\Delta }_{\mathrm{C}}(w_c,y_c) = 0$. From (18′), the equation $\mathrm{\Delta }_{\mathrm{H}}(w,y)=0$ defines the isocline $G(w,y)=0$, i.e. the curve $W_G$. Similarly the equation $\mathrm{\Delta }_{\mathrm{C}}(w,y)=0$ implicitly defines a curve $W_F$. Thus $(w_c,y_c)$ is a critical point iff it is a crossing point of the curves $W_G$ and $W_F$.

The curve $W_F$ is complicated to analyze. However, examining $(15^{\prime }$b) in ${\Gamma }_{++}$ we recognize that the curve $W_C$ defined by the equation

**Figure 5:**
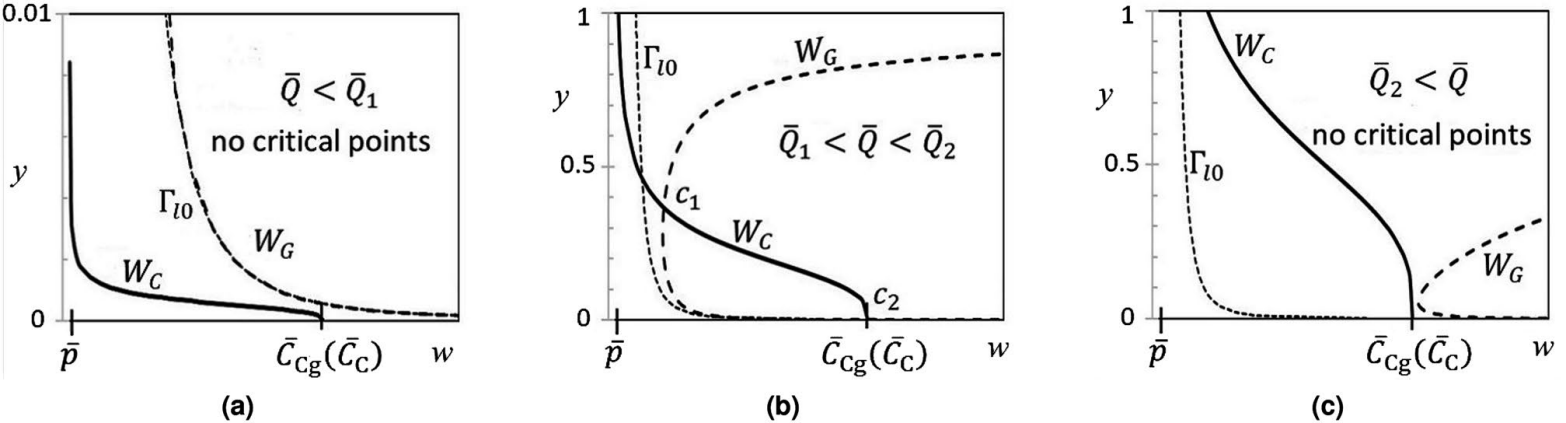
Sketches of the curves $W_C$ and $W_G$ for values of ${\overline{Q}}$ in the three ranges (**a**) ${\overline{Q}}<{\overline{Q}}_1$; (**b**) ${\overline{Q}}\in ({\overline{Q}}_1, {\overline{Q}}_2)$ and (**c**) ${\overline{Q}}_2<{\overline{Q}}$. In (**a**), for clarity, only a subset of the *y*-axis range is shown. Note that $W_G$ approaches very close to, but always lies above, the curve ${\Gamma }_{l\mathrm{0}}$.

25 $$\begin{aligned} {\Delta }^\prime _{\mathrm{C}}(w,y) = ({\overline{C}}_{\mathrm{C}}-{\overline{C}}_{\mathrm{C}{ l}}(w)){\overline{Q}}-w{\overline{Q}}_{\mathrm{g}}(w,y)=0, \end{aligned}$$

also crosses the curve $W_G$ at exactly the same critical points. Equation (25) can be explicitly solved for $y|_{W_C}$,

26 $$\begin{aligned} (y|_{W_C})^3=\left( {\overline{C}}_{\mathrm{C}}-\dfrac{w}{{\overline{K}}_{\mathrm{C}}-w} \right) \dfrac{1}{w(w-{\overline{p}})}\dfrac{{\overline{Q}}}{\overline{{\Lambda }}_{\mathrm{g}}}. \end{aligned}$$

We are only interested in the form of $W_C$ within ${\Gamma }_{++}$.

By examining the behavior of the curves $W_G$ and $W_C$ with respect to ${\overline{Q}}$, it is straightforward to determine that there exist values ${\overline{Q}}_1$ and ${\overline{Q}}_2$ such that, for ${\overline{Q}}\in ({\overline{Q}}_1, {\overline{Q}}_2)$, $W_G$ and $W_C$ cross at two critical points $c_1=(w_{c1},y_{c1})$ and $c_2=(w_{c2},y_{c2})$ in ${\Gamma }_{++}$. At each of the values ${\overline{Q}}={\overline{Q}}_1$ and ${\overline{Q}}={\overline{Q}}_2$, $W_G$ and $W_C$ touch tangentially at a single critical point. And for ${\overline{Q}}<{\overline{Q}}_1$ or ${\overline{Q}}_2<{\overline{Q}}$ there are no critical points. A sketch of this behavior is given in Fig. 5. Critical points $c_1$ and $c_2$ are also shown in Fig. 3, which is drawn for the case ${\overline{Q}}_1<{\overline{Q}}<{\overline{Q}}_2$.

#### Critical point classification

A critical point is classified by analyzing the solution of the linearized form of (18′) in the local neighborhood of equilibrium^16, 20^. With $u=w-w_c$, $v=y-y_c$ denoting the components of a small perturbation from the critical point, the linearized system is

27 $$\begin{aligned} \begin{pmatrix}\frac{du}{dt}\\ \frac{dv}{dt}\end{pmatrix}&=\begin{pmatrix}\frac{dF}{dw}&{}\frac{dF}{dy}\\ \frac{dG}{dw}&{}\frac{dG}{dy}\end{pmatrix}_{{{w_c,y_c}}}\begin{pmatrix} u\\ v\end{pmatrix}\nonumber \\ \nonumber \\&=\begin{pmatrix}\frac{F_2}{F_1}\frac{\partial \mathrm{\Delta }_{\mathrm{H}}}{\partial w}+\frac{1}{F_1}\frac{\partial {\Delta }_{\mathrm{C}}}{\partial w}\quad \qquad &{}\frac{F_2}{F_1}\frac{\partial \mathrm{\Delta }_{\mathrm{H}}}{\partial y}+\frac{1}{F_1}\frac{\partial {\Delta }_{\mathrm{C}}}{\partial y}\\ -\frac{\partial {\Delta }_{\mathrm{H}}}{\partial w}&{}-\frac{\partial {\Delta }_{\mathrm{H}}}{\partial y}\end{pmatrix}_{{w_c,y_c}}\begin{pmatrix} u\\ v\end{pmatrix}. \end{aligned}$$

In (27) we have used (18′) and the fact that $\mathrm{\Delta }_{\mathrm{H}}(w_c,y_c) = \mathrm{\Delta }_{\mathrm{C}}(w_c,y_c) = 0$. The matrix in (27) has characteristic values ${\Lambda }_\pm = (A\pm \sqrt{A^2-4D})/2$ where *A* and *D* are, respectively, the trace and determinant of the matrix in (27). Characterization of the critical points in terms of *A* and *D* is well known^16, 20^.

### Solution trajectories

#### Single-phase flow trajectory

In the single-phase flow regime, the dynamical system variable *y* satisfies $y(t)=0$. From (13) we have the relation $w={\overline{K}}_{\mathrm{C}}{\overline{C}}_{\mathrm{C}{ l}}/(1+{\overline{C}}_{\mathrm{C}{} { l}})$. We use this relation to extend the definition of the variable *w* into the single-phase regime. Then (2) provides the time development of *w*(*t*) during single phase flow.

#### Transition to two-phase flow; initial condition for gas bubble formation

An initial condition $(w_0,y_0)$ is needed to solve the two-phase flow system (18′). This is set by the initial size of the gas bubble, $y_0$, and its initial $\hbox {CO}_2$ concentration, $w_0$. Initial bubble size is determined by microscopic, non-linear dynamics at a nucleation site^9^ that are beyond the scope of this paper. In order to perform numerical computation using a transition from the single-phase to the two-phase region we determine initial bubble formation time, size and $\hbox {CO}_2$ concentration using approximations outlined in Appendix F. We recognize that conditions (F.1)–(F.3) provide an approximation to actual bubble formation. While we use these conditions in some of our numerical solutions, we also explore other bubble trajectories under the recognition that our initial conditions may not be sufficiently accurate. We note that unless ($w_0,y_0$) lies on a trajectory that either starts in, or enters, the region $G(w,y)>0$, the bubble size will monotonically decrease to 0 (the bubble dissolves).

**Figure 6:**
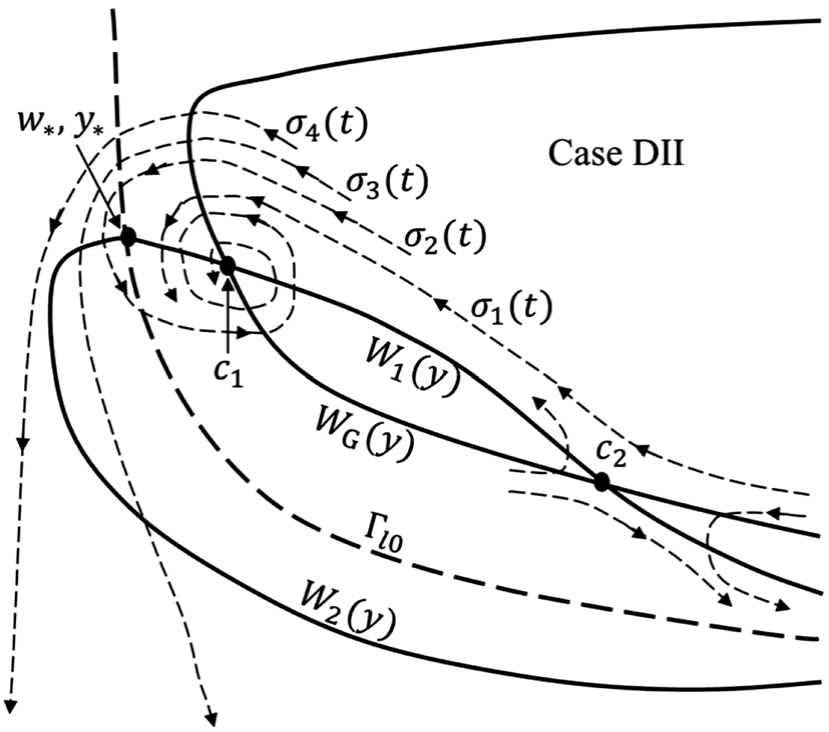
Illustration of possible trajectory behaviors in the vicinity of ($w_*,y_*$) when the critical point $c_1$ is a spiral point and $c_2$ is a saddle. There are four trajectory behaviors, $\sigma _k(t)$, $k=1,\ldots ,4$, illustrated for $W_2(y)$ having the form in case DII.

**Figure 7:**
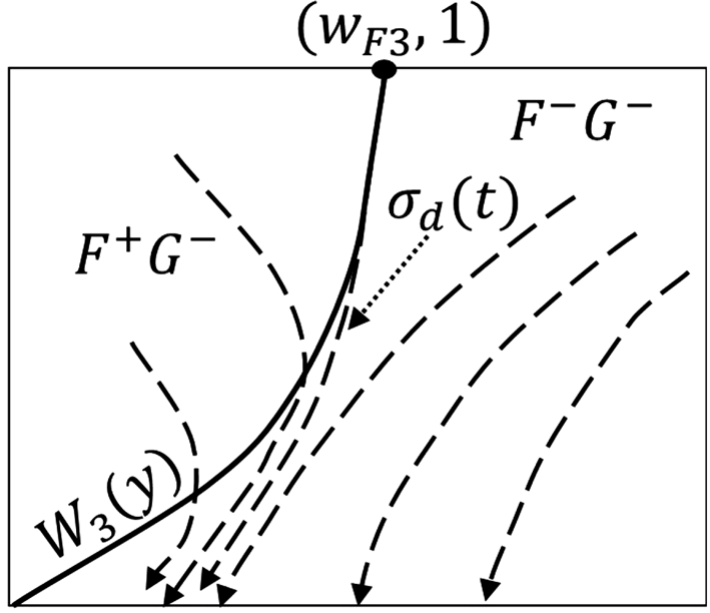
Sketch of solution trajectories in ${\Gamma }_{--}$. All solution trajectories must exit the $F^+G^-$ region. The solution trajectory $\sigma _d(t)$ separates $F^+G^-$ trajectories from all remaining trajectories in the $F^-G^-$ region.

#### Solution trajectories in ${{\Gamma }_{++}}$ and ${{\Gamma }_{-+}}$

The sign of the direction field *F*(*w*, *y*) changes across the curves $W_1(y)$, $W_2(y)$, $W_3(y)$; the sign of *G*(*w*, *y*) changes across the curve $W_G(y)$. The critical points $c_1$ and $c_2$ play a decisive role in trajectory behaviors. In our numerical computations we show that, as ${\overline{Q}}$ increases, $c_1$ changes type: from an unstable node, to an unstable spiral point and then to a stable spiral point; while $c_2$ always remains a saddle point.

For purposes of illustration of solution trajectories we consider the case when $c_1$ is a stable spiral point and $c_2$ is a saddle point. Let $\sigma (t)=(w(t), y(t))$, $w(0)=w_0$, $y(0)=y_0$, be the trajectory of a solution of (18′), where the initial condition *w*(0), *y*(0) is assumed to lie in the region ${\Gamma }_{++}$. There are four general patterns for the behavior of $\sigma (t)$. These are illustrated in Fig. 6 which is drawn for $W_2(y)$ having the form of case DII in Appendix D. The first behavior, illustrated by the trajectory labeled $\sigma _1(t)$, is that the trajectory remains in the region of ${\Gamma }_{++}$ and spirals into the critical point $c_1$. The remaining patterns shown involve those trajectories that cross the curve ${\Gamma }_{l\mathrm{0}}$. The second trajectory type, $\sigma _2(t)$, does not escape the zone of attraction of the spiral point; it enters the region $F^+G^-$ in ${\Gamma }_{-+}$, re-enters ${\Gamma }_{++}$, and spirals into the critical point $c_1$. The third, $\sigma _3(t)$, is outside of the zone of attraction; after it enters the region $F^+G^-$ in ${\Gamma }_{-+}$, it never leaves ${\Gamma }_{-+}$, ultimately entering the region $F^-G^-$ and subsequently reaches the *w*-axis. The last behavior, illustrated by $\sigma _4(t)$, enters and remains in the region $F^-G^-$ after crossing ${\Gamma }_{l\mathrm{0}}$; subsequently reaching the *w*-axis.

For trajectories $\sigma _1(t)$ and $\sigma _2(t)$, the gas bubble in the pore approaches a steady-state size. For trajectories $\sigma _3(t)$ and $\sigma _4(t)$ the bubble first grows to a maximum size before decreasing and ultimately dissolving completely. (See the section below that discusses how trajectories approach the *w*-axis.)

#### Solution trajectories ${{\Gamma }_{--}}$

From the signs of the direction fields on each side of $W_3(y)$ (Fig. 7) we deduce the following behaviors. No trajectory in the $F^-G^-$ region to the right of $W_3(y)$ can enter the $F^+G^-$ region to the left of $W_3(y)$, and any trajectory that starts in the $F^+G^-$ region must exit. In particular, the trajectory labelled $\sigma _d(t)$ in Fig. 7 that passes through the point ($w_{\text{ F3 }},1$) acts as a separator between trajectories leaving the region $F^+G^-$ and those remaining in the region $F^-G^-$.

#### Solution trajectories approaching the *w*-axis

Finally, we address the slope of trajectories approaching the $w-$axis. From (18′) and (22) it is straightforward to show that, in the regions ${\Gamma }_{--}$ and ${\Gamma }_{-+}$,

$$\begin{aligned} \lim _{y\rightarrow 0}\dfrac{G(w,y)}{F(w,y)}=\dfrac{{\overline{K}}_{\mathrm{C}}}{w(1-{\overline{K}}_{\mathrm{C}}+w)({\overline{K}}_{\mathrm{C}}-w)}. \end{aligned}$$

Thus the slope, *dy*/*dw*, of a trajectory approaches a finite, *w*-dependent value as $y\rightarrow 0$. Consequently, solution trajectories like $\sigma _3(t)$ and $\sigma _4(t)$ in Fig. 6 will evolve toward the *w*-axis. For such a trajectory, $\sigma (t)=(w(t),y(t))$, there will therefore be a time, $t_d$, such that $y(t_d )=0$ (i.e. the bubble is completely dissolved) and $w(t_d)$ is under-saturated. Since ${\overline{C}}_{\mathrm{Cg}}({\overline{C}}_{\mathrm{C}})$ is greater than $w(t_d)$, *w*(*t*) will then increase (under single-phase flow conditions) for $t>t_d$ until *w*(*t*) reaches a critical saturation value (Appendix F of the supplementary material), a new bubble will form, and a new trajectory cycle will commence.

## Numerical computations

Using Maple, numerical computations were performed to verify and illustrate our analytic results. The numerical values of all pore, physical and fluid parameters used in the computations are summarized in Table 1. With these parameter values, (24) sets a flow range limit of ${\overline{Q}}_{\mathrm{lo}} = 7.19379\times 10^{-5} \le {\overline{Q}} \le {\overline{Q}}_{\mathrm{hi}} = 3.41059\times 10^3$. However, the discussion in the section on critical point existence states that critical points can only exist for flows between ${\overline{Q}}_1$ and ${\overline{Q}}_2$ (where ${\overline{Q}}_2 = {\overline{Q}}_{\mathrm{max}}$). From the data in Table 1, we have ${\overline{Q}}_1 = 1.6\times 10^{-3}$ and ${\overline{Q}}_2 = 9.61673\times 10^{3}$. Therefore we have the relative sizes ${\overline{Q}}_{\mathrm{lo}}< {\overline{Q}}_1< {\overline{Q}}_{\mathrm{hi}} < {\overline{Q}}_2 = {\overline{Q}}_{\mathrm{max}}$. Note that two critical points always exist for the flow range ${\overline{Q}}_1 < {\overline{Q}}_{\mathrm{hi}}$ (see Fig. 5b). We perform our numerical computations in this range.

**Table 1:** Parameters and values used in the numerical computations.

$\mathrm{R}_{\mathrm{pore}}$	$5\times 10^{-3}$ cm	*V*	$5.236\times 10^{-7}$ cm$^3$
*a*	$10^{-3}$ cm	*L*	$5\times 10^{-3}$ cm
*T*	323.15 K	*R*	$8.31441\times 10^7$ dyne cm mol$^{-1}\,$K$^{-1}$
$\nu _{{ l}}$	$5.4648\times 10^{-3}$ dyne s cm$^{-2}$	$\nu _{\mathrm{g}}$	$1.6132\times 10^{-4}$ dyne s cm$^{-2}$
${\Lambda }_l$	$1.4372\times 10^{-8}$ cm$^5\,$dyne$^{-1}\,$s$^{-1}$	${\Lambda }_{\mathrm{g}}$	$4.86854\times 10^{-6}$ cm$^5$ dyne$^{-1}$ s$^{-1}$
$K_{\mathrm{C}}$	$2.2623\times 10^7$ dyne cm$^{-2}$	$K_{\mathrm{H}}$	$4.1339\times 10^4$ dyne cm$^{-2}$
$\gamma$	99.0640 dyne cm$^{-1}$	*s*	$3.96256\times 10^4$ dyne cm$^{-2}$
*p*	$10^7$ dyne cm$^{-2}$	$C_c$	$4.8\times 10^{-2}$ mol

### Critical point changes and flow regimes

**Figure 8:**
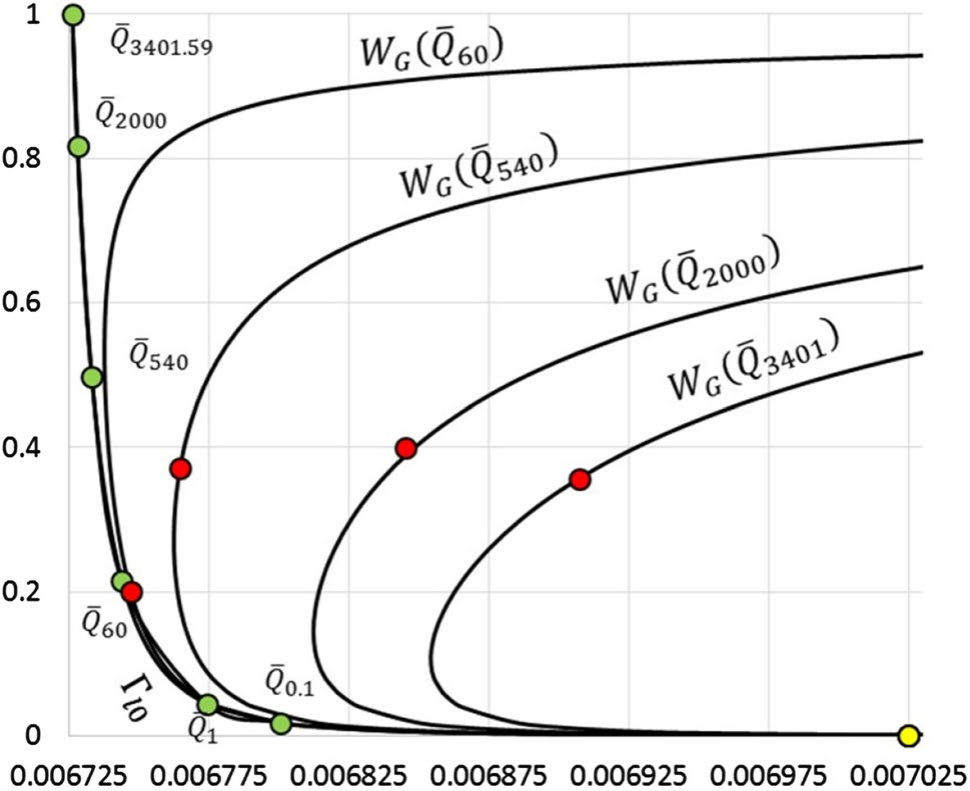
Movement of the points $w_*,y_*$ (green), $c_1$ (red), and $c_2$ (yellow) with ${\overline{Q}}$. The flow rate ${\overline{Q}}_{\mathrm{value}}$ labels the point $w_*,y_*$ as it moves on the ${\Gamma }_{l\mathrm{0}}$ curve. The critical points $c_1$ and $c_2$ are shown on their respective $W_G$ curves, which are also labeled by the appropriate flow rate.

We first examine the ${\overline{Q}}$ dependence of the character of the critical point using the linearized model (27). We find three flow regimes: R1: ${\overline{Q}}_1 \le {\overline{Q}} < 14.6431$; R2: $14.6431 \le {\overline{Q}} < 70.7865$ and R3: $70.7865 \le {\overline{Q}} \le {\overline{Q}}_{\mathrm{hi}}$. In R1 the critical point $c_1$ is an unstable node; in R2 it is an unstable spiral; and in R3 it is a stable spiral. In all regimes $c_2$ is a saddle point. Figure 8 shows the movement of the point $w_*,y_*$ and the critical points $c_1$ and $c_2$ as a function of ${\overline{Q}}$. We make the following observations.

(O1) The huge difference in scales between the *w*- and *y*-axes distorts perception of angles. Fig. G (supplementary material) shows a properly scaled view of angles in the vicinity of $c_2$ for ${\overline{Q}}=494$.

(O2) The curve $W_G$ closely approaches ${\Gamma }_{l\mathrm{0}}$, both in the vicinity of $c_2$ and as ${\overline{Q}}$ decreases. This is evident in Fig. 8 and Fig. G (Appendix G).

(O3) The critical point $c_2$ moves very slowly with ${\overline{Q}}$, remaining in the close vicinity of the point $({\overline{C}}_{\mathrm{Cg}}({\overline{C}}_{\mathrm{C}})$, $y_{{\tiny l0}}({\overline{C}}_{\mathrm{C}}))$ with $w_{c2}\le {\overline{C}}_{\mathrm{Cg}}({\overline{C}}_{\mathrm{C}})$, $y_{c2} > y_{{\tiny l0}}({\overline{C}}_{\mathrm{C}})$. At the value ${\overline{Q}}={\overline{Q}}_{\mathrm{max}}$, the critical points $c_1$ and $c_2$ coalesce on the $w = {\overline{C}}_{\mathrm{Cg}}({\overline{C}}_{\mathrm{C}})$ boundary.

### Gas bubble dynamics

To detail the evolution of gas bubbles, we performed computations using the nonlinear system (18′) for a value of ${\overline{Q}}$ from each regime. The first computation is for regime R3 using the value ${\overline{Q}}=475$.

**Figure 9:**
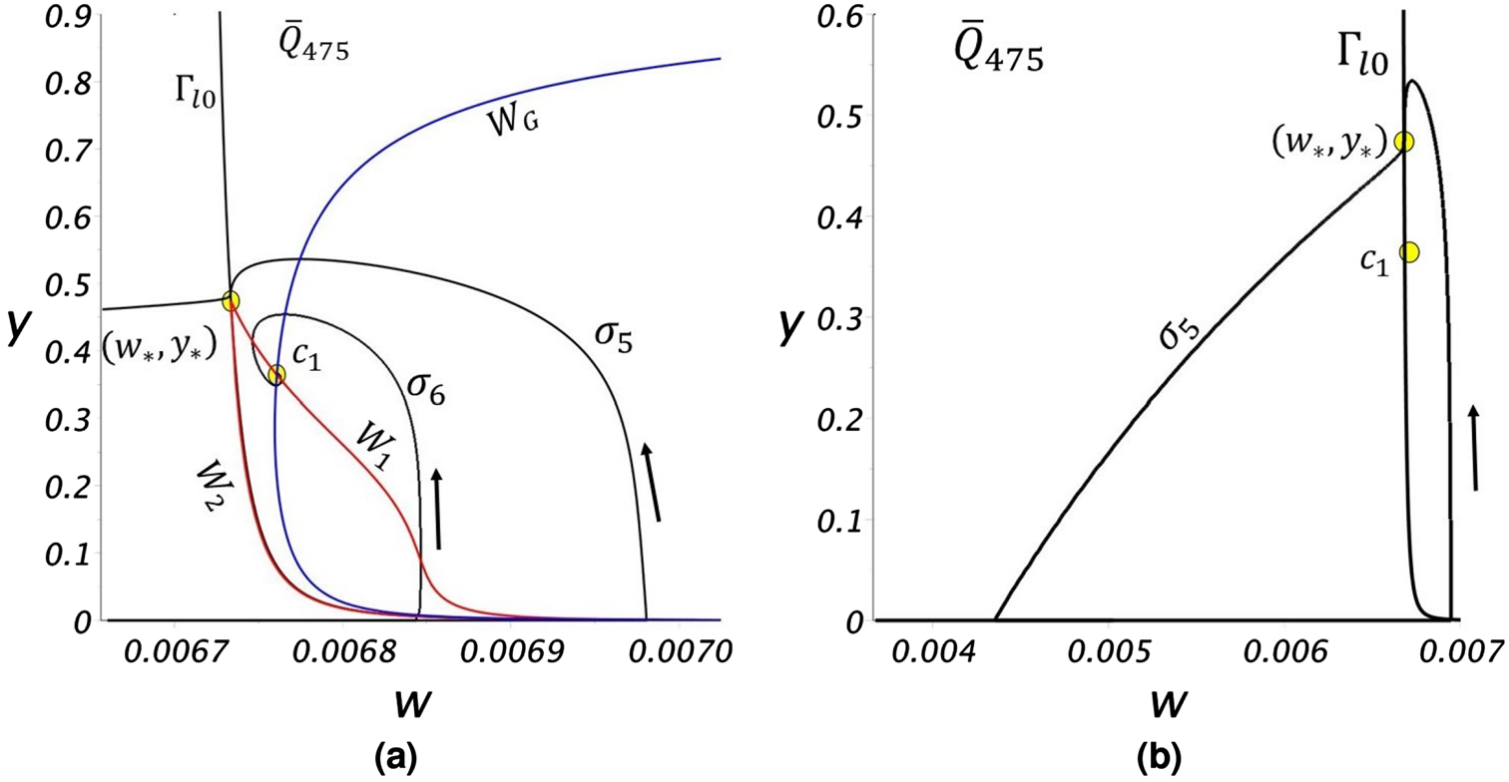
(**a**) A view of two solution trajectories for ${\overline{Q}}=475$. For trajectory $\sigma _5$, $(w_0,y_0)=(6.9806\times 10^{-3},8\times 10^{-3})$ and for trajectory $\sigma _6$, $(w_0,y_0)=(6.8437\times 10^{-3},8\times 10^{-3})$. (**b**) A view showing one complete cycle of $\sigma _5$.

Figure 9a shows a portion of a phase-space trajectory ($\sigma _5$). For this value of ${\overline{Q}}$, using the bubble-point equations (F.1)–(F.3) the pore reaches critical saturation at $t=8.3296\times 10^{-3}$. A gas bubble forms in ${\Gamma }_{++}$ with $(w_0,y_0)=(6.9806\times 10^{-3},8\times 10^{-3})$. The gas bubble growth trajectory crosses ${\Gamma }_{l\mathrm{0}}$ at a time $t_1=8.6718\times 10^{-3}$ at the point $(w_{{\tiny l0}},y_{{\tiny l0}})=(6.733216\times 10^{-3},0.484318)$ where $y_{{\tiny l0}} \ge y_*$. At this point, $dw/dt < 0$ and $dy/dt < 0$, $\sigma _5$ enters ${\Gamma }_{-+}$, and evolves (Fig. 9b) to the *y*-axis with the complete dissolution of the bubble at a finite time $t_2=8.7116\times 10^{-3}$. For $t > t_2$, $\sigma _5$ follows the *w*-axis under single-phase flow conditions with *w*(*t*) increasing until, again using (F.1)–(F.3), at $t_3=1.6626\times 10^{-2}$ the trajectory reaches the bubble point $(6.9806\times 10^{-3},0)$, the gas bubble re-emerges and a new cycle begins. Fig. 9b shows the complete trajectory cycle for $\sigma _5$.

**Figure 10:**
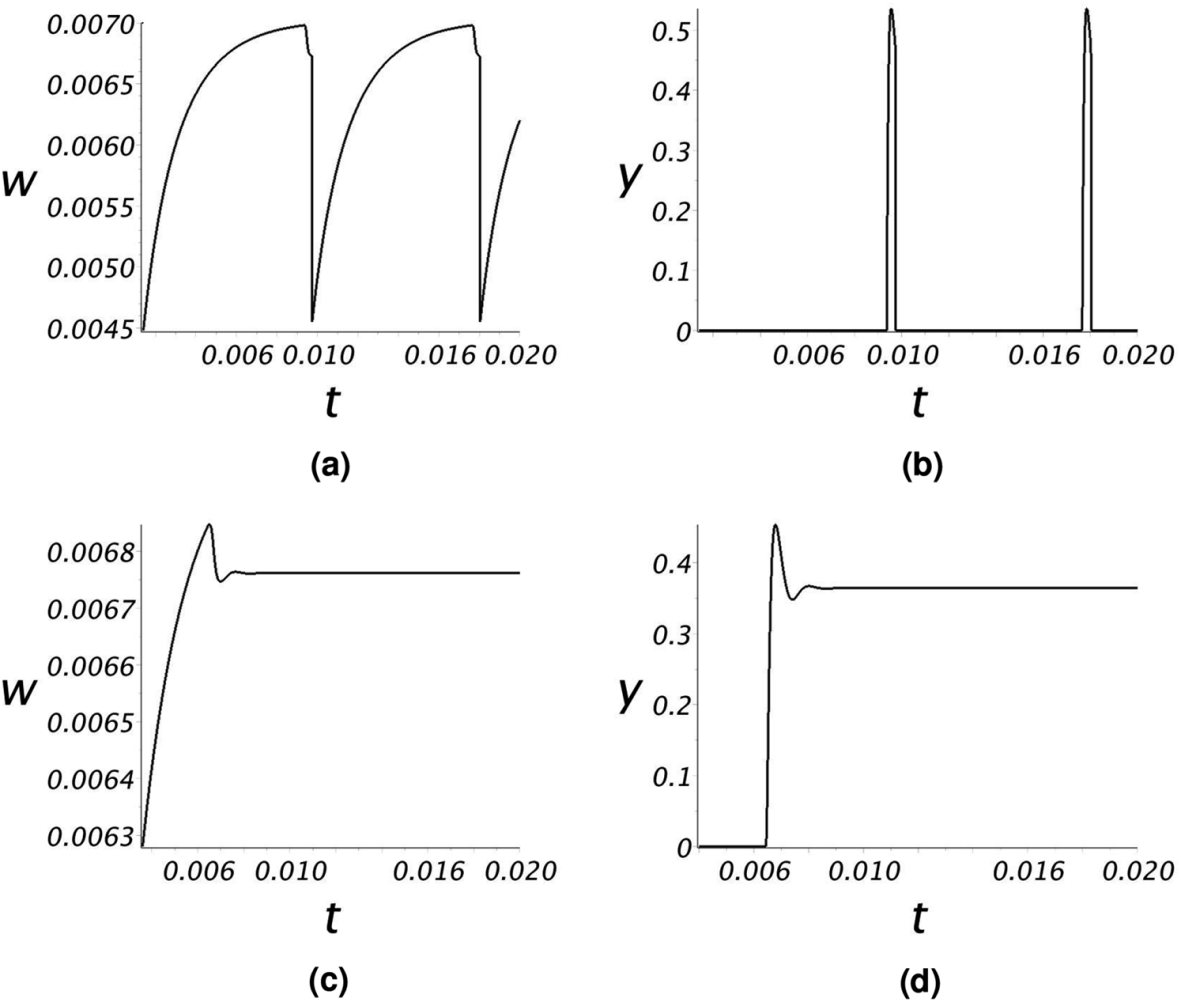
Graphs of (**a**) *w*(*t*) and (**b**) *y*(*t*) for two cycles of $\sigma _5$, and of (**c**) *w*(*t*) and (**d**) *y*(*t*) for $\sigma _6$ of Fig. 9.

Figure 10a,b plot *w*(*t*) and *y*(*t*) over two cycles of $\sigma _5$. The dissolved $\hbox {CO}_2$ concentration in the liquid phase, *w*(*t*), accumulates in the pore until the bubble point is reached. Once the bubble point is reached, time scales shorten dramatically as the bubble expands and subsequently dissolves very rapidly (Fig. 10b). Over this period, the $\hbox {CO}_2$ concentration in the liquid phase drops precipitously.

Figure 9a also shows the trajectory $\sigma _6$ for a bubble that is assumed (i.e. not using equations (F.1)–(F.3)) to form with $(w_0,y_0)=(6.8437\times 10^{-3},8\times 10^{-3})$. In this case the trajectory lies within the attractor region of $c_1$ and spirals into the critical point. The values *w*(*t*) and *y*(*t*) for $\sigma _6$ are plotted in Fig. 10c,d, documenting how the dissolved $\hbox {CO}_2$ concentration in the liquid phase and the gas phase saturation approach steady state values. Note there is a rather sharp oscillation of the bubble prior to reaching steady state.

**Figure 11:**
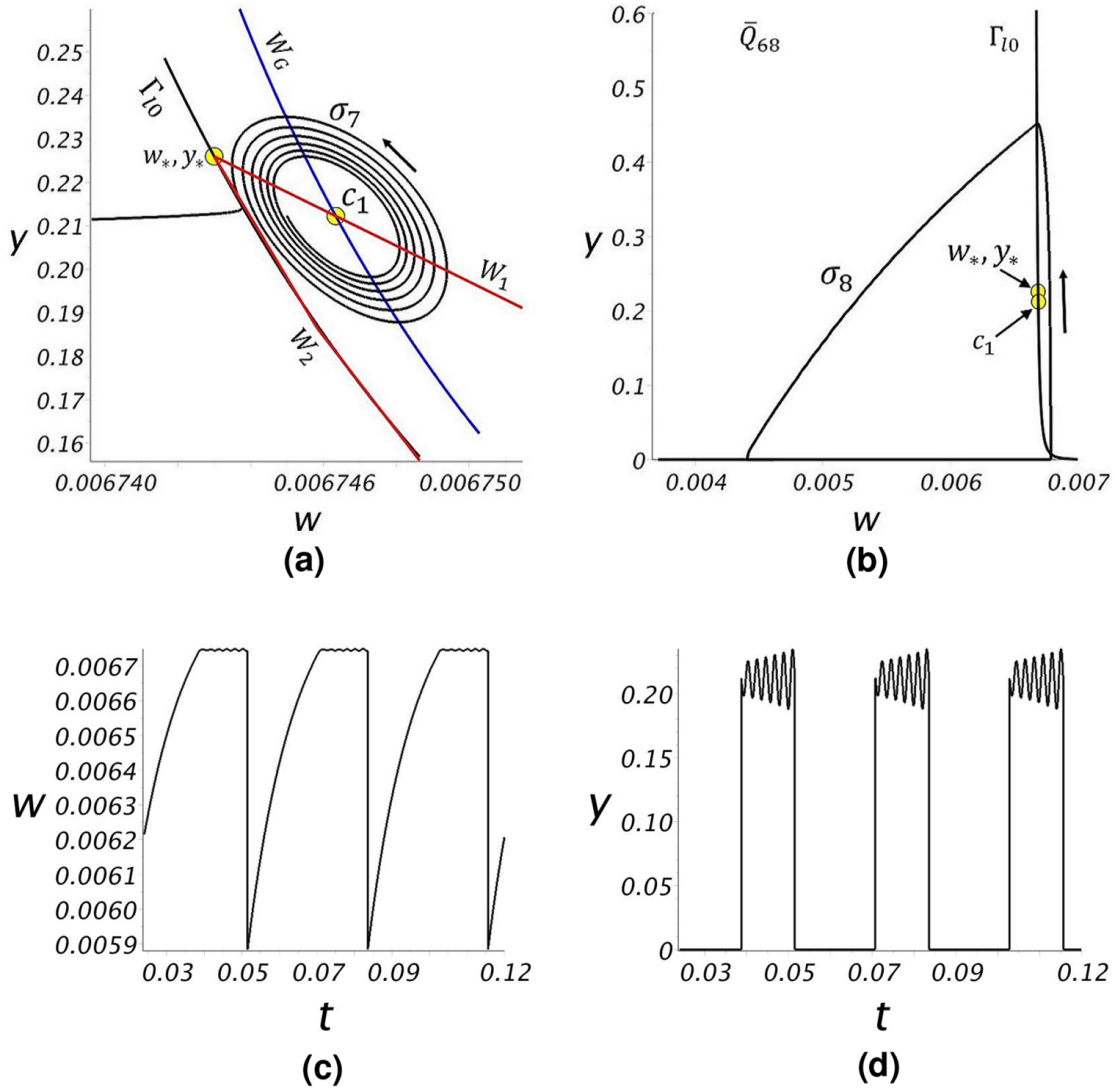
Solution trajectories for ${\overline{Q}}=68$ when (**a**) $(w_0,y_0)=(6.744\times 10^{-3},2.12\times 10^{-1})$ and (**b**) $(w_0, y_0)=(6.83\times 10^{-3},8\times 10^{-3})$. Graphs of (**c**) w(t) and (**d**) y(t) for three cycles of $\sigma _7$.

The second computations explore regime R2 using the flow rate ${\overline{Q}} = 68$. Only cyclic bubble formation occurs in this flow regime. If a bubble forms sufficiently close to the unstable spiral point, $c_1$, the cycle may involve an initial period of instability in the bubble growth (Fig. 11a). If the bubble initializes on a more “remote” trajectory, the cycle of bubble formation and dissolution is more regular (Fig. 11b). Fig. 11c,d plot *w*(*t*) and *y*(*t*) over a time range covering two cycles of the trajectory $\sigma _7$ in Fig. 11a.

The third computations explored regime R1 using the flow rate ${\overline{Q}}=13$. Only cyclic bubble formation occurs in this flow regime. Trajectories are similar to that shown for $\sigma _8$ in Fig. 11b.

## Discussion

The mathematical analysis presented in this paper, motivated and supported by our earlier network flow computations^6^, leads us to a somewhat unexpected conclusion: namely that when a gas component “bubbles-out” from a saturated solution under steady state flow in porous media, the phase space conditions under which such bubbles achieve a stable size is limited. Our results indicate that, for sufficiently low flow rates (regimes R1 and R2), any gas bubble that forms will re-dissolve, possibly (regime R2) after undergoing oscillatory behavior. In the (much wider) range of faster flow rates (regime R3), whether a gas bubble remains stable or decays depends on the fine-scale details of bubble initiation. In all cases involving bubble dissolution, under steady flow conditions the behavior of bubble formation and re-dissolution repeats cyclically.

Our numerical computations show that these regimes cover a range of conditions pertinent to reservoir flow. Therefore, with respect to flow management in reservoir operations (specifically trying to maintain multi-component, saturated, single phase flow), our results imply that, once pressure conditions allow bubble formation, there is a small “window of opportunity” at low flow rates (regimes R1 and R2) to restrict bubble formation to at most cyclic behavior. However once flow rates exceed the rather low threshold delineating regime R3, whether bubbles form stably or cyclically depends on microscopic processes beyond reservoir management control.

Our investigation is not exhaustive; we have analyzed the dynamical system (18′) by employing a number of assumptions. These assumptions are summarized in Appendix H (supplementary material) and fall into two general categories, physical (e.g. liquid phase is perfectly wetting) and technical. The technical assumptions rely on having parameter values similar to petroleum reservoirs (Table 1). We have not investigated removing these assumptions, except to make a comment regarding the flow-rate restriction (24). Two of the inequalities in (24) ensure that the point ($w_*,y_*$) on ${\Gamma }_{l\mathrm{0}}$ lies within the phase space. This restriction can be lifted to some extent, allowing ($w_*,y_*$) to move somewhat outside of the phase space. Reducing this restriction will extend the analysis to a larger range of flow values but will produce little change in the qualitative aspects of our analysis.

The *y*-dependence in the dynamical system is a critical determinant in the resulting behavior. This dependence comes from three sources: the capillary pressure term (10) where we assume $r=y^{1/3}\mathrm{R}_{\mathrm{pore}}$, the distribution of water and $\hbox {CO}_2$ mass (16) where the y-dependence is linear, and from the fractional flow functions *f*(*y*) and *g*(*y*) which introduce complicated nonlinear dependence. It is possible that different forms for the fractional flow functions could significantly alter the gas bubble behavior of this dynamical system.

One possible critique of our analysis is that bubble dissolution, which is governed by the evolution of trajectories in ${\Gamma }_{--}$ and ${\Gamma }_{-+}$ (i.e. under back-flow conditions), is solely due to our assumption that back-flow involves pure water invading the pore from the outlet reservoir. Such back-flow reduces ${\overline{C}}_{\mathrm{C}{} { l}}$ concentration in the pore, potentially forcing dissolution of the gas phase. In actual porous media flow situations, back-flow would involve water containing $\hbox {CO}_2$ (at concentrations less than ${\overline{C}}_{\mathrm{C}{} { l}}$) infiltrating the pore. Back-flow under these more realistic conditions would affect the shape of trajectories in the regions ${\Gamma }_{--}$ and ${\Gamma }_{-+}$, opening the possibility that trajectories that enter ${\Gamma }_{-+}$ from ${\Gamma }_{++}$ may re-enter ${\Gamma }_{++}$. However, changing back-flow conditions should not affect the analysis in ${\Gamma }_{++}$, and specifically not affect the type of critical points. Thus, at low flow conditions there would still be no attractor node to produce stable bubbles. At higher flow rates a stable spiral node exists, but it has a finite-sized region of attraction (as can be inferred from trajectory segments in the vicinity of the saddle $c_2$ in Fig. 6). Thus not all trajectories would lead to the growth of stable bubbles. Finally, we note that our computations in Chang and Lindquist^6^, which included realistic back-flow conditions, did produce bubble dissolution.

While we have examined the case where the gas phase is $\hbox {CO}_2$, the analysis should hold for any other gas that preserves the assumptions used. Similarly, we have considered a liquid phase consisting solely of $\hbox {H}_2$O, with dissolved $\hbox {CO}_2$. This invites the question of how applicable our analysis is to a more complicated brine phase.

## Supplementary information

Supplementary Information.
