# Supplementary material for: The dynamics of gas-bubble formation at saturated conditions in porous media flow
Source: Sci Rep. 2020 Aug 6;10:13175. doi: 10.1038/s41598-020-69506-w (PMC7413368; doi:10.1038/s41598-020-69506-w)
Supplement: Supplementary file 1 — Supplementary Information. [file 41598_2020_69506_MOESM1_ESM.pdf]

# Supplement: The Dynamics of Gas-Bubble Formation at Saturated Conditions in Porous Media Flow

**K. Alex Chang**[1,*] **and W. Brent Lindquist**[2]

[1]National Pingtung University, Department of Applied Mathematics, Pingtung, Taiwan, ROC.
[2]Texas Tech University, Departments of Mathematics & Statistics and Geosciences, Lubbock Texas, USA.
[*]chang@mail.nptu.edu.tw

## Appendix A The direction field $dy/dt = G(w,y)$

From ($18'$), ($15'$a), ($7'$a), and ($14'$) we start with the expression for the direction field $dy/dt = G(w,y)$,

$$G(w,y) = -\overline{Q} + \overline{\Lambda}_l f(y)[w - \overline{p} - \overline{s}y^{-1/3}], \tag{A.1}$$

where $f(y)$ is given by (8). It will simplify exposition (just for this appendix) to switch variables from $(w,y)$ to $(\omega,z)$ defined by $\omega = [w-\overline{p}]/\overline{s}$, $z = y^{1/3}$. The map $\omega(w)$ has the range $\omega \in [-\overline{p}/\overline{s}, \omega(\overline{C}_\mathrm{C})]$ where $\omega(\overline{C}_\mathrm{C}) \stackrel{\mathrm{def}}{=} [\overline{C}_{\mathrm{Cg}}(\overline{C}_\mathrm{C}) - \overline{p})]/\overline{s}$. The points $w = \overline{p}$ and $w = \overline{p} + \overline{s}$ correspond to the values $\omega = 0$ and $\omega = 1$ respectively. Under this change of variables, (A.1) can be written

$$\frac{G(\omega,z)}{\overline{\Lambda}_l\overline{s}} = -\frac{\overline{Q}}{\overline{\Lambda}_l\overline{s}} + f(z)[\omega - z^{-1}]. \tag{A.2}$$

We consider the isoclines of $G(\omega,z)$. Parametrizing the isoclines by the value $G = \beta\overline{Q}$, where $\beta$ is a free parameter, from (A.2) each isocline has the form

$$\omega(z;\alpha) = \frac{1}{z} + \frac{\alpha}{f(z)}, \qquad \text{where} \quad \alpha = (1+\beta)\overline{Q}/(\overline{\Lambda}_l\overline{s}). \tag{A.3}$$

The value $\alpha = 0$ (i.e. $\beta = -1$) corresponds to the curve $\Gamma_{l0}$ on which $G = -\overline{Q}$. The equation for $\Gamma_{l0}$ in $(\omega,z)$ space is $\omega(z,0) = 1/z$. The $\alpha > 0$ isoclines correspond to $\overline{Q}_l > 0$, thus they cover the region $\Gamma_{++}$, while the $\alpha < 0$ isoclines cover the regions $\Gamma_{-+}$ and $\Gamma_{--}$. From (A.3) note that all isoclines have the value $G < 0$ in $\Gamma_{-+}$ and $\Gamma_{--}$. Thus the condition $G = 0$ is only possible in the region $\Gamma_{++}$.

We first consider the $\alpha > 0$ isoclines. From (8), in $\Gamma_{++}$ $f(z)$ has the form, $f(z) = (1+z^3/2)(1-z^3)^2$. It is straightforward to show that $f(z)$ is monotonically decreasing on $z \in [0,1]$ with $f(0) = 1$, $f(1) = 0$, $f'(0) = f'(1) = 0$, $f'(z) < 0$, and $f''(z) < 0$ on $(0, 2^{-1/3})$ while $f''(z) > 0$ on $(2^{-1/3}, 1]$. Defining $h(z) = 1/f(z)$, we have

$$\omega(z;\alpha) = 1/z + \alpha h(z) > \omega(z;0), \tag{A.4}$$

which satisfies $d^2\omega(z;\alpha)/dz^2 > 0$. For $\alpha > 0$, $\omega(z;\alpha)$ is a function that is "concave up" having a unique minimum $\omega_{\min}(\alpha)$ at the point $z_{\min}(\alpha) \in (0,1)$ satisfying,

$$z_{\min}^2 h'(z_{\min}) = \alpha^{-1}, \qquad \omega_{\min}(\alpha) = 1/z_{\min} + \alpha h(z_{\min}). \tag{A.5}$$

In physical space $\omega$ is bounded above by the value $\omega(\overline{C}_\mathrm{C})$. However, viewed as a function of $z$, (A.3) implicitly extends the range of $\omega$ values comprising the $\alpha$'th islocline to $\omega(z;\alpha) \in [\omega_{\min}(\alpha), \infty]$ with $\lim_{z\to 0^+} \omega(z;\alpha) = \lim_{z\to 1^-} \omega(z;\alpha) \to \infty$. There exists some isocline value $\alpha_{\max} > 0$ such that $\omega_{\min}(\alpha_{\max}) = \omega(\overline{C}_\mathrm{C})$ and $\omega_{\min}(\alpha) < \omega(\overline{C}_\mathrm{C})$ for $0 < \alpha < \alpha_{\max}$. Consequently, from (A.4) and (A.5) we conclude there exist values $z_{\mathrm{G1}}(\alpha)$ and $z_{\mathrm{G2}}(\alpha)$ satisfying $0 < z_{\mathrm{G1}}(\alpha) < z_{\min}(\alpha) < z_{\mathrm{G2}}(\alpha) < 1$ such that

$$\omega(z_{\mathrm{G1}}(\alpha);\alpha) = \omega(z_{\mathrm{G2}}(\alpha);\alpha) = \omega(\overline{C}_\mathrm{C}) \quad \text{for} \quad \alpha < \alpha_{\max}.$$

For the isocline $\omega(z;\alpha_{\max})$, we have $z_{G1}(\alpha_{\max}) = z_{\min}(\alpha_{\max}) = z_{G2}(\alpha_{\max})$. Fig. A.1(a) provides a sketch of the isoclines $\omega(z;0)$ and $\omega(z;\alpha < \alpha_{\max})$, indicating the points $(\omega_{\min}(\alpha), z_{\min}(\alpha))$, $(\omega(\overline{C}_\mathrm{C}), z_{G1}(\alpha))$ and $(\omega(\overline{C}_\mathrm{C}), z_{G2}(\alpha))$.

The value of $\alpha_{\max}$ places restrictions on $\overline{Q}$. From (A.3) we note that the isocline $\alpha = \overline{Q}/(\overline{\Lambda}_l\overline{s})$ carries the constant value $G = 0$. Therefore if $\overline{Q}/(\overline{\Lambda}_l\overline{s}) > \alpha_{\max}$, then $G < 0$ everywhere in $\Gamma_{++}$ (and therefore everywhere in the physical phase space).

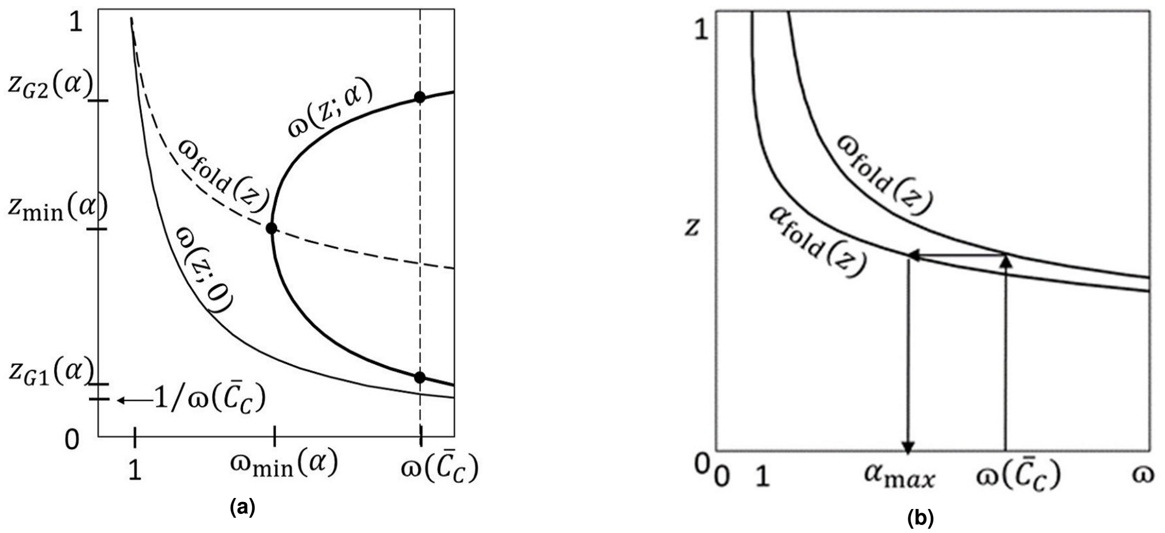


**Figure A.1.** (a) Plots of $\Gamma_{l0} = \omega(z;0)$ and example isocline $\omega(z;\alpha)(\alpha < \alpha_{\max})$ showing the fold curve $\omega_{\text{fold}}(z)$ and the values $z_{G1}(\alpha)$, $z_{\min}(\alpha)$, $z_{G2}(\alpha)$, $\omega_{\min}(\alpha)$, and $\omega(\overline{C}_C)$. (b) Plots of the curves (A.7a) and (A.8) and an illustration of the computation needed to determine the value $\alpha_{\max}$.

Consequently, the direction field $dy/dt$ would be negative everywhere in the physical phase space and no critical point could exist. Under these conditions, any gas bubble that tries to form dissolves rapidly and the physical phase space will not support gas bubbles. Therefore $\overline{Q}$ must be sufficiently small such that

$$\overline{Q} < \alpha_{\max}\overline{\Lambda}_l\overline{s} \stackrel{\text{def}}{=} \overline{Q}_{\max}. \tag{A.6}$$

To compute $\alpha_{\max}$ note that, as $\alpha$ increases from the value 0, the points $(z_{\min}(\alpha), \omega_{\min}(\alpha))$ trace out a curve. This curve defines a "fold" in the function $G(\omega, z)$ across which $G$ achieves maximum values in the $z$ (but not $\omega$) direction. The parameter $\alpha$ can be eliminated from the two equations in (A.5) giving the equation for this fold curve,

$$\omega_{\text{fold}}(z) = \frac{1}{z} + \frac{h(z)}{z^2 h'(z)} = \frac{2}{9}\frac{(1+2z^3)^2}{z^4(1+z^3)}. \tag{A.7a}$$

Note that $\omega_{\text{fold}}(z) > 0$ on $[0,1]$ with $\omega_{\text{fold}}(1) = 1$. In $w$, $y$ coordinates, the fold curve will be denoted

$$W_{\text{fold}}(y) = \overline{p} + \overline{s}\frac{2}{9}\frac{(1+2y)^2}{y^{4/3}(1+y)}. \tag{A.7b}$$

A sketch of the curve $\omega_{\text{fold}}(z)$ is also provided in Fig. A.1(a).

Inverting the first equation in (A.5) gives the values of $\alpha$ along the fold curve $\omega_{\text{fold}}(z)$,

$$\alpha_{\text{fold}}(z) = \frac{1}{z^2 h'(z)} = \frac{2}{9}\frac{(1+z^3/2)^2(1-z^3)^3}{z^4(1+z^3)}. \tag{A.8}$$

Note that the right-hand sides of (A.7a) and (A.8) are only functions of $z$ (i.e. of $y = s_g$), independent of any other physical parameter in the problem. Thus the two curves $\omega_{\text{fold}}(z)$ and $\alpha_{\text{fold}}(z)$ can be computed, independent of any particular flow problem in this geometry. These two curves are plotted in Fig. A.1(b). This figure illustrates how $\alpha_{\max}$ is computed and how the physical parameters ($\overline{K}_C$, $\overline{C}_C$, $\overline{p}$, $\overline{s}$ and $\overline{\Lambda}_l$) enter through the value $w(\overline{C}_C)$ and the computation (A.6) for $\overline{Q}_{\max}$.

With the value of $\overline{Q}$ restricted by (A.6), the isocline $G(w,y) = 0$ exists in $\Gamma_{++}$. In the text we shall refer to this isocline as the curve $W_G(y)$ (see Fig. A.2(a)), and the two values where this curve intersects the boundary $w = C_{Cg}(\overline{C}_C)$ will be denoted $y_{G1}$ and $y_{G2}$, where $y_{G1} < y_{G2}$.

We will have need of the partial derivatives $\partial G(w,y)/\partial w$ and $\partial G(w,y)/\partial y$. In $\Gamma_{++}$ these can be computed directly from

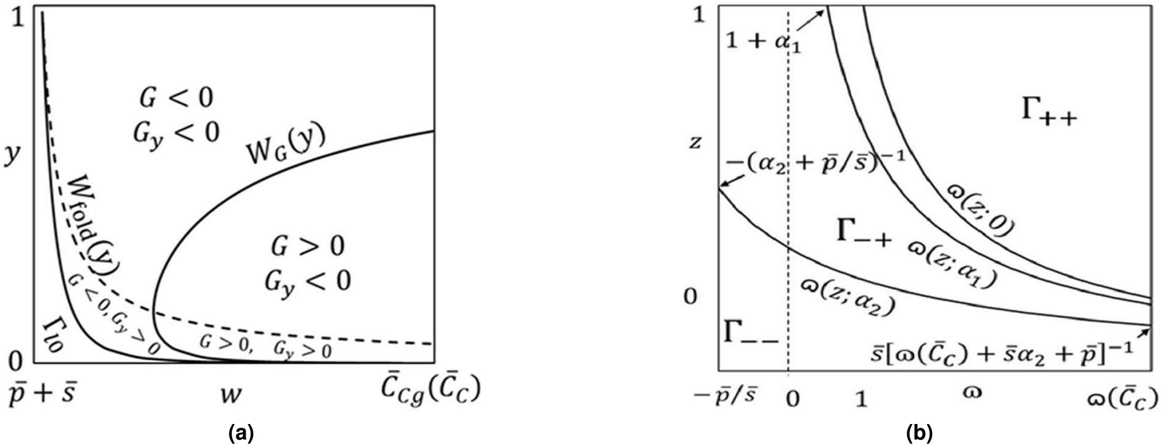


**Figure A.2.** (a) Variation of the sign of $G(w,y)$ and its partial derivative $G_y$ in $\Gamma_{++}$. Note that the curves $\Gamma_{l0}$ and $W_{\mathrm{G}}(y)$ may approach very close, but do not cross. (b) Example $G(\omega,z)$ isoclines in $\Gamma_{-+}$ and $\Gamma_{--}$, showing axis intersection values.

(A.1):

$$\frac{\partial G(w,y)}{\partial w} = \overline{\Lambda}_l f(y) \begin{cases} >0 & \text{on} \quad y \in [0,1), \\ =0 & \text{for} \quad y = 1, \end{cases}$$

$$\frac{\partial G(w,y)}{\partial y} = \overline{\Lambda}_l f'(y) \left\{ w - \overline{p} - \overline{s} \left[ \frac{1}{y^{1/3}} - \frac{f(y)}{3f'(y)y^{3/4}} \right] \right\} = -\frac{3}{2}\overline{\Lambda}_l (1-y)^2 [w - W_{\mathrm{fold}}(y)].$$

Note that $\partial G(w,y)/\partial y = 0$ on the curves $y = 1$ and $W_{\mathrm{fold}}(y)$. Thus when crossing the curve $W_{\mathrm{fold}}(y)$, the partial $\partial G(w,y)/\partial y$ changes sign, while the sign of the partial $\partial G(w,y)/\partial w$ remains unchanged. Fig. A.2(a) illustrates the respective signs of $G(w,y)$ and its partial derivative $G_y$ in $\Gamma_{++}$.

We now consider the form of the isoclines for $\alpha < 0$ in the regions $\Gamma_{-+}$ and $\Gamma_{--}$. With $f(z) = 1$, the equation of each isocline is simply

$$\omega(z;\alpha) = \frac{1}{z} + \alpha. \tag{A.9}$$

Fig. A.2(b) plots two of these isoclines showing relevant intersection points with the physical axes. Note that as $\alpha \to -\infty$, the isoclines approach the $z = 0$ axis.

The needed partial derivatives of $G(w,y)$ in $\Gamma_{-+}$ and $\Gamma_{--}$ are straightforward to compute from (A.1) (with $f(y) = 1$),

$$\frac{\partial G(w,y)}{\partial w} = \overline{\Lambda}_l > 0, \qquad \frac{\partial G(w,y)}{\partial y} = \frac{\overline{\Lambda}_l \overline{s}}{3y^{4/3}} > 0.$$

There is no change in the signs of $G(w,y)$ or its partial derivatives $G_w$, $G_y$ across the curves $\Gamma_{l0}$ or $\Gamma_{\mathrm{g}0}$.

## Appendix B $\widetilde{F}(w,y)$ on $\Gamma_{l0}$

We address the sign of $\widetilde{F}(w,y)$ along the curve $\Gamma_{l0}$. We begin with the form of $\widetilde{F}(w,y)$ in (23b) which we write as

$$\widetilde{F}(w,y)|_{\Gamma_{l0}} = \overline{\Lambda}_{\mathrm{g}} \overline{s}(\overline{K}_{\mathrm{C}} - w)w \left[ \frac{\overline{Q}}{\overline{\Lambda}_{\mathrm{g}}\overline{s}} \left( 1 + \frac{\overline{C}_{\mathrm{C}}}{w} - \frac{1}{\overline{K}_{\mathrm{C}} - w} \right) - \left( \frac{\overline{s}}{w - \overline{p}} \right)^8 \right]$$
$$\stackrel{\mathrm{def}}{=} H_1(w) \left[ H_2(w) - \left( \frac{\overline{s}}{w - \overline{p}} \right)^8 \right]. \tag{B.10}$$

Our interest is in the domain $\overline{p} + \overline{s} \le w \le \overline{C}_{\mathrm{Cg}}(\overline{C}_{\mathrm{C}})$. Here $H_1(w) > 0$. From restriction (24) we note that

$$\frac{\overline{Q}}{\overline{\Lambda}_{\mathrm{g}}\overline{s}} \left( 1 + \frac{\overline{C}_{\mathrm{C}}}{\overline{p} + \overline{s}} - \frac{1}{\overline{K}_{\mathrm{C}} - \overline{p} - \overline{s}} \right) < 1, \qquad \frac{\overline{Q}}{\overline{\Lambda}_{\mathrm{g}}\overline{s}} > \left( \frac{\overline{s}}{\overline{C}_{\mathrm{Cg}}(\overline{C}_{\mathrm{C}}) - \overline{p}} \right)^8. \tag{B.11}$$

**Lemma B.** *Given* (B.11) *and the assumption*

$$\frac{4\overline{K}_C}{(1+\overline{C}_C+\overline{K}_C)^2} \ll 1, \tag{B.12}$$

*then* $\widetilde{F}(w,y)|_{\Gamma_{I0}} = 0$ *at a single point* $w_*$, $y_* = y_{I0}(w_*)$ *where* $\overline{p}+\overline{s} < w_* < \overline{C}_{Cg}(\overline{C}_C)$.

**Proof:** We show that the functions $H_2(w)$ and $y_{I0}(w)^{8/3} = [\overline{s}/(w-\overline{p})]^8$ in (B.10) intersect at a single value $w_*$ in the interval $(\overline{p}+\overline{s}, \overline{C}_{Cg}(\overline{C}_C))$. We proceed by elucidating the properties of $H_2(w)$ and $y_{I0}(w)^{8/3}$ needed for this proof. For $H_2(w)$ we have the following observations:

$$\begin{aligned} &\lim_{w\to 0^+} H_2(w) \to \infty; && \lim_{w\to \overline{K}_C^-} H_2(w) \to -\infty; \\ &H_2(w_+) = 0, \quad \text{where} && w_+ \approx \overline{C}_{Cg}(\overline{C}_C+\overline{K}_C) \text{ and } \overline{C}_{Cg}(\overline{C}_C) < w_+ < \overline{K}_C. \end{aligned} \tag{B.13}$$

To verify (B.13), note from (B.10) that $H_2(w) = 0$ for $w$ satisfying $w^2 + (\overline{C}_C+\overline{K}_C)w - \overline{C}_C\overline{K}_C = 0$. Solving this quadratic for the positive root under assumption (B.12) we have, to first order in a Taylor expansion of the square-root of the quadratic discriminant,

$$w_+ \approx \frac{\overline{K}_C(\overline{C}_C+\overline{K}_C)}{1+\overline{C}_C+\overline{K}_C}.$$

The first two derivatives of $H_2(w)$ are

$$H_2'(w) = -\frac{\overline{Q}}{\Lambda_g\overline{s}}\left[\frac{\overline{C}_C}{w^2} + \frac{1}{(\overline{K}_C-w)^2}\right] < 0, \qquad H_2''(w) = 2\frac{\overline{Q}}{\Lambda_g\overline{s}}\left[\frac{\overline{C}_C}{w^3} - \frac{1}{(\overline{K}_C-w)^3}\right]. \tag{B.14}$$

From (B.14), $H_2''(w)$ must have a single root in the interval $w \in (0, \overline{K}_C)$. Since $\lim_{w\to 0^+} H_2''(w) > 0$ and

$$\begin{aligned} H_2''(\overline{C}_{Cg}(\overline{C}_C)) &= 2\frac{\overline{Q}}{\Lambda_g\overline{s}}\left[\frac{\overline{C}_C}{(\overline{C}_{Cg}(\overline{C}_C))^3} - \frac{1}{(\overline{K}_C-\overline{C}_{Cg}(\overline{C}_C))^3}\right] \\ &= 2\frac{\overline{Q}}{\Lambda_g\overline{s}}\frac{\overline{C}_C(1-\overline{C}_C^2)}{(\overline{C}_{Cg}(\overline{C}_C))^3} > 0, \end{aligned} \tag{B.15}$$

then the single root of $H_2''(w)$ must lie between $\overline{C}_{Cg}(\overline{C}_C)$ and $\overline{K}_C$. In (B.15) we have used the fact that $\overline{C}_C < 1$. Thus from (B.14) and (B.15) we have

$$H_2''(w) > 0 \text{ on } (0, \overline{C}_{Cg}(\overline{C}_C)). \tag{B.16}$$

For $y_{I0}(w)^{8/3}$ we have the observations that, for $w > 0$,

$$\frac{d}{dw}(y_{I0}(w))^{8/3} = -8(y_{I0}(w))^3\,\overline{s}^{-1} < 0, \tag{B.17a}$$

$$\frac{d^2}{dw^2}(y_{I0}(w))^{8/3} = 72(y_{I0}(w))^{10/3}\,\overline{s}^{-2} > 0. \tag{B.17b}$$

Equations (B.14), (B.16) and (B.17) state that $H_2(w)$ and $y_{I0}(w)^{8/3}$ are both monotonic, decreasing, concave-up functions on the interval $(0, \overline{C}_{Cg}(\overline{C}_C))$. In addition (B.11) guarantees that $H_2(\overline{p}+\overline{s}) < y_{I0}(\overline{p}+\overline{s})^{8/3}$ and $H_2(\overline{C}_{Cg}(\overline{C}_C)) > y_{I0}(\overline{C}_{Cg}(\overline{C}_C))^{8/3}$. Thus we have the existence of a single value $w_*$ in the interval $(\overline{p}+\overline{s}, \overline{C}_{Cg}(\overline{C}_C))$ such that $H_2(w_*) = y_{I0}(w_*)^{8/3}$, i.e. such that $\widetilde{F}(w_*, y_{I0}(w_*)) = 0$. Let $y_* \stackrel{\text{def}}{=} y_{I0}(w_*)$. ■

As a consequence of Lemma B, we have

$$\widetilde{F}(w, y_{I0}(w)) \begin{cases} < 0, & w \in [\overline{p}+\overline{s}, w_*), \\ = 0, & w = w_*, \\ > 0, & w \in (w_*, \overline{C}_{Cg}(\overline{C}_C)]. \end{cases} \tag{B.18}$$

## Appendix C $\widetilde{F}(w,y)$ in $\Gamma_{++}$

We address the variation of the sign of $\widetilde{F}(w,y)$ in $\Gamma_{++}$ by analyzing (23a). We begin with the behavior of $\widetilde{F}(w,y)$ on the boundaries of $\Gamma_{++}$. The behavior of $\widetilde{F}(w,y)$ on the boundary $\Gamma_{l0}$ was analyzed in Appendix B. On the boundary $y = 1$,

$$\widetilde{F}(w,1) = \overline{\Lambda}_{\rm g}\overline{s}(\overline{K}_{\rm C} - w)(F_2(w) + \overline{C}_{\rm C})\left\{\frac{\overline{Q}}{\overline{\Lambda}_{\rm g}\overline{s}} - \frac{w(w-\overline{p})}{(F_2(w)+\overline{C}_{\rm C})\overline{s}}\right\}. \tag{C.19}$$

On this boundary, $\overline{p} + \overline{s} \le w \le \overline{C}_{\rm Cg}(\overline{C}_{\rm C}) < \overline{K}_{\rm C}$; therefore the term $(\overline{K}_{\rm C} - w)(F_2(w) + \overline{C}_{\rm C})$ is positive. In addition,

$$\frac{\overline{Q}}{\overline{\Lambda}_{\rm g}\overline{s}} < \frac{(\overline{p}+\overline{s})}{(F_2(\overline{p}+\overline{s})+\overline{C}_{\rm C})}\frac{(\overline{p}+\overline{s}-\overline{p})}{\overline{s}} < \frac{w(w-\overline{p})}{(F_2(w)+\overline{C}_{\rm C})\overline{s}}, \tag{C.20}$$

where the first inequality in (C.20) comes from (24). By (C.20), $\widetilde{F}(w,1) < 0$ for $\overline{p}+\overline{s} \le w \le \overline{C}_{\rm Cg}(\overline{C}_{\rm C}) < \overline{K}_{\rm C}$.

The final boundary is $w = \overline{C}_{\rm Cg}(\overline{C}_{\rm C})$, $y \in (y_{l0}(\overline{C}_{\rm C}), 1)$. Using (18′) and (15′), we can derive an expression relating $\widetilde{F}(w,y)$ and $G(w,y)$,

$$\widetilde{F}(w,y) = (\overline{K}_{\rm C} - w)\{-[F_2(w)+\overline{C}_{\rm C}]G(w,y) + (\overline{C}_{\rm C} - \overline{C}_{{\rm C}l}(w))\overline{Q}_l(w,y) - w\overline{Q}_{\rm g}(w,y)\}.$$

This expression simplifies considerably when evaluated at $w = \overline{C}_{\rm Cg}(\overline{C}_{\rm C})$,

$$\widetilde{F}(\overline{C}_{\rm Cg}(\overline{C}_{\rm C}),y) = -\frac{\overline{K}_{\rm C}^2\overline{C}_{\rm C}}{(1+\overline{C}_{\rm C})^2}\left\{G(\overline{C}_{\rm Cg}(\overline{C}_{\rm C}),y) + \overline{Q}_{\rm g}(\overline{C}_{\rm Cg}(\overline{C}_{\rm C}),y)\right\}. \tag{C.21}$$

We note that $\overline{Q}_{\rm g}(\overline{C}_{\rm Cg}(\overline{C}_{\rm C}),y) > 0$. From (C.21) and the results on the signs of $G(w,y)$ summarized in Fig. A.2(a), we have

$$\widetilde{F}(\overline{C}_{\rm Cg}(\overline{C}_{\rm C}),y) < 0 \quad \text{for} \quad y \in [y_{\rm G1}, y_{\rm G2}]. \tag{C.22}$$

From (B.18) we have

$$\widetilde{F}(\overline{C}_{\rm Cg}(\overline{C}_{\rm C}), y_{l0}(\overline{C}_{\rm C})) > 0. \tag{C.23}$$

Taking the derivative of (C.21) with respect to $y$ yields

$$\frac{d\widetilde{F}(\overline{C}_{\rm Cg}(\overline{C}_{\rm C}),y)}{dy} = -\frac{\overline{K}_{\rm C}^2\overline{C}_{\rm C}}{(1+\overline{C}_{\rm C})^2}\left\{\frac{dG(\overline{C}_{\rm Cg}(\overline{C}_{\rm C}),y)}{dy} + 3\overline{\Lambda}_{\rm g}\left(\overline{C}_{\rm Cg}(\overline{C}_{\rm C}) - \overline{p}\right)y^2\right\}. \tag{C.24}$$

From (C.24) and the results on the signs of the partial $\partial G/\partial y$ summarized in Fig. A.2(a), we conclude

$$\frac{d\widetilde{F}(\overline{C}_{\rm Cg}(\overline{C}_{\rm C}),y)}{dy} < 0 \quad \text{for} \quad y \in [y_{l0}(\overline{C}_{\rm C}), y_{\rm G1}]. \tag{C.25}$$

Therefore (C.22), (C.23) and (C.25) imply there exists a single value $y_{\rm F1}$ such that

$$\widetilde{F}(\overline{C}_{\rm Cg}(\overline{C}_{\rm C}), y_{\rm F1}) = 0 \;\text{ with }\; y_{l0}(\overline{C}_{\rm Cg}(\overline{C}_{\rm C})) < y_{\rm F1} < y_{\rm G1}. \tag{C.26}$$

From (C.22) and the discussion relative to (C.19) we know $\widetilde{F}(\overline{C}_{\rm Cg}(\overline{C}_{\rm C}), y_{\rm G2}) < 0$ and $\widetilde{F}(\overline{C}_{\rm Cg}(\overline{C}_{\rm C}),1) < 0$. If $y_* \le y_{\rm G2}$, the results from cases C1 and C2 discussed below lead to the conclusion that $\widetilde{F}(\overline{C}_{\rm Cg}(\overline{C}_{\rm C}),y) < 0$ for $y \in [y_{\rm G2}, 1]$. However if $y_* > y_{\rm G2}$, there exists the possibility that $\widetilde{F}(\overline{C}_{\rm Cg}(\overline{C}_{\rm C}),y)$ has an even number of roots on the interval $y \in (y_{\rm G2}, y_*)$. Evaluating (C.24) for parameter values typical to those in Table 1 we can demonstrate that $d\widetilde{F}(\overline{C}_{\rm Cg}(\overline{C}_{\rm C}),y)/dy < 0$ for $y \in [y_{\rm G2}, 1]$; consequently $\widetilde{F}(\overline{C}_{\rm Cg}(\overline{C}_{\rm C}),y)$ has no zero on $y \in [y_{\rm G2}, 1]$. Summarizing the results on this boundary we have

$$\widetilde{F}(\overline{C}_{\rm Cg}(\overline{C}_{\rm C}),y)\begin{cases} <0, & y \in (y_{\rm F1}, 1], \\ =0, & y = y_{\rm F1}, \\ >0, & y \in [y_{l0}(\overline{C}_{\rm Cg}(\overline{C}_{\rm C})), y_{\rm F1}). \end{cases} \tag{C.27}$$

We now consider the behavior of $\widetilde{F}(w,y)$ in the interior of $\Gamma_{++}$. We begin by explicitly isolating the $w$ dependence of $\widetilde{F}(w,y)$. From (23a)

$$\widetilde{F}(w,y) = \overline{K}_{\rm C}\overline{C}_{\rm C}\overline{Q} + c_1(y)w + c_2(y)w^2 + (\overline{\Lambda}_l f(y) + \overline{\Lambda}_{\rm g} g(y))w^3, \tag{C.28a}$$

where

$$c_1(y) = (\overline{K}_C - \overline{C}_C - 1)\overline{Q} + \overline{K}_C\overline{p}(\overline{\Lambda}_l f(y) + \overline{\Lambda}_g g(y)) + \overline{K}_C\overline{s}\overline{\Lambda}_l f(y) y^{-1/3}, \tag{C.28b}$$

$$c_2(y) = -\overline{Q} - (\overline{K}_C + \overline{p})(\overline{\Lambda}_l f(y) + \overline{\Lambda}_g g(y)) - \overline{s}\overline{\Lambda}_l f(y) y^{-1/3}. \tag{C.28c}$$

For any fixed value of $y \in (y_{l0}(\overline{C}_C), 1)$ we examine the behavior of (C.28a) as a function of $w$ over the interval $(-\infty, \infty)$. We have

$$\lim_{w\to-\infty} \widetilde{F}(w,y) \to -\infty, \qquad \widetilde{F}(0,y) > 0, \qquad \lim_{w\to\infty} \widetilde{F}(w,y) \to \infty. \tag{C.29a}$$

From (23a) we have

$$\lim_{w\to\overline{K}_C^-} \widetilde{F}(w,y)|_{\Gamma_{++}} = -w\overline{Q} < 0. \tag{C.29b}$$

Equations (C.28a) and (C.29) specify that $\widetilde{F}(w,y)$ has three real roots, $w_1(y)$, $w_2(y)$, $w_3(y)$, where we assign $-\infty < w_1(y) < 0$ and $\overline{K}_C < w_3(y) < \infty$. Before proceeding to determine the root $w_2(y)$, we note the following lemma.
**Lemma C.** *$y_* > y_{F1}$, where $y_*$ is defined in* Lemma B *and $y_{F1}$ is defined in* (C.26).
**Proof:** Assume $y_* \le y_{F1}$. First consider the case $y_* < y_{F1}$. Let $y$ be a fixed value lying in the interval $(y_*, y_{F1})$. By (B.18), $\widetilde{F}(w_{l0}(y), y) < 0$. We therefore conclude that the root $w_2(y)$ lies in the interval $(0, w_{l0}(y))$. However, $\widetilde{F}(\overline{C}_{Cg}(\overline{C}_C), y) > 0$ implying there is a fourth root of $\widetilde{F}(w,y)$ lying in the interval $(w_{l0}(y), \overline{C}_{Cg}(\overline{C}_C))$. Since $\widetilde{F}(w,y)$ is a polynomial of degree 3 in $w$, this is impossible. Therefore, the case $y_* < y_{F1}$ is untenable. For the case $y_* = y_{F1}$, $\widetilde{F}(w_*, y_*) = \widetilde{F}(\overline{C}_{Cg}(\overline{C}_C), y_{F1}) = 0$, again implying the existence of at least four real roots. Thus we conclude $y_* > y_{F1}$. ■

Note that Lemma C implies the relationship $y_{F1}(y_*)$ which must satisfy $y_{F1} \to y_{l0}(\overline{C}_C)$ as $y_* \to y_{l0}(\overline{C}_C)$ while maintaining the inequality $y_* > y_{F1}$.

In determining $w_2(y)$, we examine all possible cases on the fixed value of $y \in (y_{l0}(\overline{C}_{Cg}(\overline{C}_C)), 1)$.
**Case C1**. Let $y$ lie in the interval $(y_*, 1)$. By (B.18), $\widetilde{F}(w_{l0}(y), y) < 0$ and we conclude that the root $w_2(y)$ lies in the interval $(0, w_{l0}(y))$.
**Case C2**. Let $y = y_*$. Then $\widetilde{F}(w_{l0}(y_*), y_*) = 0$ and we identify $w_2(y_*) = w_{l0}(y_*)$. (As noted above, if $y_* \le y_{G2}$, cases C1 and C2 lead to the conclusion that $\widetilde{F}(\overline{C}_{Cg}(\overline{C}_C), y) < 0$ for $y \in [y_{G2}, 1]$.)
**Case C3**. Let $y$ lie in the interval $(y_{F1}, y_*)$. By (B.18), $\widetilde{F}(w_{l0}(y), y) > 0$. From (C.27) $\widetilde{F}(\overline{C}_{Cg}(\overline{C}_C), y) < 0$, and we conclude $w_2(y)$ lies in the interval $(w_{l0}(y), \overline{C}_{Cg}(\overline{C}_C))$.
**Case C4**. Let $y = y_{F1}$. By (B.18), $\widetilde{F}(w_{l0}(y), y) > 0$. From (C.27), $\widetilde{F}(\overline{C}_{Cg}(\overline{C}_C), y_{F1}) = 0$ and we conclude $w_2(y) = \overline{C}_{Cg}(\overline{C}_C)$.
**Case C5**. Let $y$ lie in the interval $(y_{l0}(\overline{C}_{Cg}(\overline{C}_C)), y_{F1})$. By (B.18), $\widetilde{F}(w_{l0}(y), y) > 0$. From (C.27), $\widetilde{F}(\overline{C}_{Cg}(\overline{C}_C), y) > 0$. Therefore, $\overline{C}_{Cg}(\overline{C}_C) < w_2(y) < \overline{K}_C$. (Note we have assigned the root $w_3(y)$ to lie in $(\overline{K}_C, \infty)$.)

Cases C1 through C5 imply that $\widetilde{F}(w,y)|_{\Gamma_{++}} = 0$ only on a curve segment $W_1(y)$ traced out by the root $w_2(y)$ for $y \in [y_*, y_{F1}]$. A sketch of $W_1(y)$, which joins the point $(w_*, y_*)$ to $(\overline{C}_{Cg}(\overline{C}_C), y_{F1})$, is shown in Fig. 3 in the text.

## Appendix D $\widetilde{F}(w,y)$ in $\Gamma_{-+}$

We address the variation of the sign of $\widetilde{F}(w,y)$ in $\Gamma_{-+}$ by analyzing (23c). We start with the behavior of $\widetilde{F}(w,y)$ on the boundaries of $\Gamma_{-+}$. On the boundary $y = 0$, $w \in [\overline{p}, \overline{C}_{Cg}(\overline{C}_C)]$,

$$\lim_{y\to 0^+} \widetilde{F}(w,y) \to \overline{\Lambda}_l \overline{s}(\overline{K}_C - w) F_2(w) y^{-1/3} \to -\infty. \tag{D.30}$$

From (23d) and restriction (24) we have demonstrated in the paper that $\widetilde{F}(w,y) < 0$ on the boundary $\Gamma_{g0}$. On the boundary $y = 1$, $w \in [\overline{p}, \overline{p} + \overline{s}]$,

$$\widetilde{F}(w,1) = \overline{K}_C\overline{C}_C\overline{Q} + d_1 w + d_2 w^2 + (\overline{\Lambda}_l + \overline{\Lambda}_g) w^3, \tag{D.31}$$

where

$$d_1 = -(1 - \overline{K}_C + \overline{C}_C)\overline{Q} - \overline{\Lambda}_l(1 - \overline{K}_C)(\overline{p} + \overline{s}) + \overline{\Lambda}_g\overline{K}_C\overline{p},$$
$$d_2 = -\overline{Q} + \overline{\Lambda}_l(1 - \overline{K}_C - \overline{p} - \overline{s}) - \overline{\Lambda}_g(\overline{K}_C + \overline{p}).$$

Considering (D.31) as a function of $w$ on $(-\infty,\infty)$ we note

$$\begin{aligned} &\lim_{w\to-\infty}\widetilde{F}(w,1)\to-\infty, \quad \lim_{w\to\infty}\widetilde{F}(w,1)\to\infty, \\ &\widetilde{F}(0,1)>0, \quad \widetilde{F}(\overline{p},1)<0, \quad \widetilde{F}(\overline{p}+\overline{s},1)<0. \end{aligned} \tag{D.32}$$

The last two inequalities in (D.32) follow from (24). Equations (D.32) imply that $\widetilde{F}(w,1)$ has exactly three real roots, $w_1$, $w_2$, $w_3$, satisfying $w_1<0<w_2<\overline{p}<\overline{p}+\overline{s}<w_3$. We therefore conclude $\widetilde{F}(w,1)<0$ on $[\overline{p},\overline{p}+\overline{s}]$.

The behavior of $\widetilde{F}(w,y)$ on the boundary $\Gamma_{l0}$ was analyzed in Appendix B. For the boundary segment $w=\overline{C}_{\mathrm{Cg}}(\overline{C}_{\mathrm{C}})$, $y\in(0,y_{l0}(\overline{C}_{\mathrm{Cg}}(\overline{C}_{\mathrm{C}})))$, we know $\widetilde{F}(\overline{C}_{\mathrm{Cg}}(\overline{C}_{\mathrm{C}}),y_{l0}(\overline{C}_{\mathrm{Cg}}(\overline{C}_{\mathrm{C}})))>0$ while $\lim_{y\to0^+}\widetilde{F}(\overline{C}_{\mathrm{Cg}}(\overline{C}_{\mathrm{C}}),y)\to-\infty$. Thus, there exists at least one point $y_{\mathrm{F2}}$ on this boundary interval such that $\widetilde{F}(\overline{C}_{\mathrm{Cg}}(\overline{C}_{\mathrm{C}}),y_{\mathrm{F2}})=0$. We assert here that $y_{\mathrm{F2}}\in(0,y_{l0}(\overline{C}_{\mathrm{Cg}}(\overline{C}_{\mathrm{C}})))$ is a unique value on this boundary interval satisfying

$$\widetilde{F}(\overline{C}_{\mathrm{Cg}}(\overline{C}_{\mathrm{C}}),y)\begin{cases} >0, & y\in(y_{\mathrm{F2}},y_{l0}(\overline{C}_{\mathrm{Cg}}(\overline{C}_{\mathrm{C}}))), \\ =0, & y=y_{\mathrm{F2}}, \\ <0, & y\in(0,y_{\mathrm{F2}}). \end{cases} \tag{D.33}$$

This assertion is proven below as part of the analysis (cases DI and DII) on the dependence of $\widetilde{F}(w,y)|_{\Gamma_{-+}}$ on $y$.

We consider the behavior of $\widetilde{F}(w,y)|_{\Gamma_{-+}}$ in the interior of $\Gamma_{-+}$ by first examing its dependence along lines of constant $y$. From (23c),

$$\widetilde{F}(w,y)=\overline{K}_{\mathrm{C}}\overline{C}_{\mathrm{C}}\overline{Q}+d_1(y)w+d_2(y)w^2+(\overline{\Lambda}_l+\overline{\Lambda}_{\mathrm{g}}g(y))w^3, \tag{D.34a}$$

where

$$d_1=c_1(y)|_{f(y)=1}-\overline{\Lambda}_l\left(\overline{p}+\overline{s}y^{-1/3}\right), \qquad d_2(y)=c_2(y)|_{f(y)=1}+\overline{\Lambda}_l, \tag{D.34b}$$

with $c_1(y)$, $c_2(y)$ given by (C.28). We examine all possible cases (labelled D0a-f) on the fixed value of $y\in(0,1)$.

**D0a:** For any fixed value $y_1\in(y_*,1)$, from (D.34a),

$$\begin{aligned} &\lim_{w\to-\infty}\widetilde{F}(w,y_1)\to-\infty, \quad \lim_{w\to\infty}\widetilde{F}(w,y_1)\to\infty, \\ &\widetilde{F}(0,y_1)>0 \quad \widetilde{F}(\overline{p},y_1)<0, \quad \widetilde{F}(w_{l0}(y_1),y_1)<0. \end{aligned} \tag{D.35}$$

Equations (D.34a) and (D.35) specify that $\widetilde{F}(w,y_1)$ has only three real roots, $w_1(y_1)$, $w_2(y_1)$, $w_3(y_1)$, where $w_1(y_1)<0<w_2(y_1)<\overline{p}$ and $w_{l0}(y_1)<w_3(y_1)$. Thus $\widetilde{F}(w,y_1)\neq0$ for any $(w,y_1>y_*)\in\Gamma_{-+}$.

**D0b:** If $y_1=y_*$, then (D.35) holds with the last equation changed to $\widetilde{F}(w_*,y_*)=0$ and we conclude the third real root satisfies $w_3(y_1)=w_*$.

**D0c:** If $y_1\in[y_{l0}(\overline{C}_{\mathrm{Cg}}(\overline{C}_{\mathrm{C}})),y_*)$, then (D.35) holds with the last equation changed to $\widetilde{F}(w_{l0}(y_1),y_1)>0$ and we conclude $\overline{p}<w_3(y_1)<w_{l0}(y_1)$.

**D0d:** If $y_1\in(y_{F2},y_{l0}(\overline{C}_{\mathrm{Cg}}(\overline{C}_{\mathrm{C}})))$, then (D.35) holds with the last equation changed to $\widetilde{F}(\overline{C}_{\mathrm{Cg}}(\overline{C}_{\mathrm{C}}),y_1)>0$ and we conclude $\overline{p}<w_3(y_1)<\overline{C}_{\mathrm{Cg}}(\overline{C}_{\mathrm{C}})$.

**D0e:** If $y_1=y_{\mathrm{F2}}$, then (D.35) holds with the last equation changed to $\widetilde{F}(\overline{C}_{\mathrm{Cg}}(\overline{C}_{\mathrm{C}}),y_1)=0$ and we conclude $w_3(y_1)=\overline{C}_{\mathrm{Cg}}(\overline{C}_{\mathrm{C}})$.

**D0f:** If $y_1\in(0,y_{\mathrm{F2}})$, then (D.35) holds with the last equation changed to $\widetilde{F}(\overline{C}_{\mathrm{Cg}}(\overline{C}_{\mathrm{C}}),y_1)<0$ and we conclude $\overline{C}_{\mathrm{Cg}}(\overline{C}_{\mathrm{C}})<w_3(y_1)$. Thus $\widetilde{F}(w,y_1)\neq0$ for any point $(w,y_1<y_{\mathrm{F2}})\in\Gamma_{-+}$.

As $\widetilde{F}(w,y)|_{\Gamma_{-+}}$ is a continuous function in both of its variables, cases D0a through D0f imply that $\widetilde{F}(w,y)|_{\Gamma_{-+}}=0$ only on a continuous curve segment $W_2(y)$ traced out by the root $w_3(y)$ for $y\in[y_{\mathrm{F2}},y_*]$. $W_2(y)$ joins the point $(w_*,y_*)$ to the point $(\overline{C}_{\mathrm{Cg}}(\overline{C}_{\mathrm{C}}),y_{\mathrm{F2}})$. Note $W_2(y)$ is a single-valued function of $y$.

To further explore the behavior of the curve $W_2(y)$ we examine the $y$-dependence of $\widetilde{F}(w,y)|_{\Gamma_{-+}}$ along lines of constant $w$. From (23c) the first two partial derivatives of $\widetilde{F}(w,y)|_{\Gamma_{-+}}$ with respect to $y$ are

$$\frac{\partial\widetilde{F}(w,y)}{\partial y}=(\overline{K}_{\mathrm{C}}-w)\left\{-F_2(w)\frac{\overline{\Lambda}_l\overline{s}}{3y^{4/3}}-3\overline{\Lambda}_{\mathrm{g}}w(w-\overline{p})y^2\right\}, \tag{D.36a}$$

$$\frac{\partial^2\widetilde{F}(w,y)}{\partial y^2}=(\overline{K}_{\mathrm{C}}-w)\left\{F_2(w)\frac{4\overline{\Lambda}_l\overline{s}}{9y^{7/3}}-6\overline{\Lambda}_{\mathrm{g}}w(w-\overline{p})y\right\}<0. \tag{D.36b}$$

In obtaining the inequality in (D.36b), recall that $F_2(w) < 0$. For any fixed value of $w \in (\overline{p}, \overline{C}_{\mathrm{Cg}}(\overline{C}_{\mathrm{C}})]$, $\widetilde{F}(w,y)|_{\Gamma_{-+}}$ is concave down having maximum value at $y_{\max}(w)$ where the first partial, $(\partial \widetilde{F}(w,y)/\partial y$, vanishes. From (D.36a), $y_{\max}(w)$ satisfies

$$(y_{\max}(w))^{10/3} = \frac{-F_2(w)\overline{s}\delta}{w(w-\overline{p})} = \frac{\overline{s}\delta[1-(\overline{K}_{\mathrm{C}}-w)]}{(w-\overline{p})(\overline{K}_{\mathrm{C}}-w)}, \qquad \overline{p} < w < \overline{K}_{\mathrm{C}}, \tag{D.37}$$

where $\delta \equiv \overline{\Lambda}_l/(9\overline{\Lambda}_{\mathrm{g}})$. Note, $y_{\max}(w) > 0$ for $w \in (\overline{p}, \overline{K}_{\mathrm{C}})$. As a function of $w$, the curve (D.37) has the following limits: $\lim_{w\to\overline{p}^+} y_{\max}(w) \to \infty$ and $\lim_{w\to\overline{K}_{\mathrm{C}}^-} y_{\max}(w) \to \infty$. From (D.37), $y_{\max}(w) = 1$ at two values, $\widehat{w}_\pm$, satisfying the quadratic

$$\widehat{w}_\pm^2 - (\overline{K}_{\mathrm{C}} + \overline{p} - \overline{s}\delta)\widehat{w}_\pm + \overline{K}_c\overline{p} + \overline{s}\delta(1-\overline{K}_{\mathrm{C}}) = 0.$$

Assuming (see e.g. Table 1) that $4\overline{s}\delta \ll (\overline{K}_{\mathrm{C}} + \overline{p} - \overline{s}\delta)^2$, to first order in a Taylor expansion of the square root in the quadratic formula solution we have,

$$\widehat{w}_\pm \cong \frac{1}{2}\left\{(\overline{K}_{\mathrm{C}} + \overline{p} - \overline{s}\delta) \pm (\overline{K}_{\mathrm{C}} - \overline{p} + \overline{s}\delta)\left[1 - \frac{2\overline{s}\delta}{(\overline{K}_{\mathrm{C}} - \overline{p} + \overline{s}\delta)^2}\right]\right\}.$$

Evaluating these two values we see

$$\widehat{w}_- \cong \overline{p} + \overline{s}\delta\frac{1-(\overline{K}_{\mathrm{C}} - \overline{p} + \overline{s}\delta)}{\overline{K}_{\mathrm{C}} - \overline{p} + \overline{s}\delta} \gtrsim \overline{p}, \quad \widehat{w}_+ \cong \overline{K}_{\mathrm{C}} - \frac{\overline{s}\delta}{\overline{K}_{\mathrm{C}} - \overline{p} + \overline{s}\delta} \lesssim \overline{K}_{\mathrm{C}}.$$

Thus only $\widehat{w}_-$ lies in the region $\Gamma_{-+}$. We rename this value as $\widehat{w}_- \stackrel{\text{def}}{=} \widehat{w}_1$.

We next consider the local extremum values of the curve $y_{\max}(w)$ at which

$$\frac{dy_{\max}(w)}{dw} = \frac{3}{10}[y_{\max}(w)]^{-7/3}\,\overline{s}\delta\frac{[(\overline{K}_{\mathrm{C}} - w)^2 + 2w - \overline{K}_{\mathrm{C}} - \overline{p}]}{(\overline{K}_{\mathrm{C}} - w)^2\,(w-\overline{p})^2} = 0.$$

The extrema occur at two values $\breve{w}_\pm$ satisfying $(\overline{K}_{\mathrm{C}} - \breve{w}_\pm)^2 - 2(\overline{K}_{\mathrm{C}} - \breve{w}_\pm) + \overline{K}_{\mathrm{C}} - \overline{p} = 0$, i.e. $\breve{w}_\pm = \overline{p} - (1-\overline{K}_{\mathrm{C}} + \overline{p}) \pm (1-\overline{K}_{\mathrm{C}} + \overline{p})^{1/2}$. Noting that $\overline{p} < \overline{K}_{\mathrm{C}} < 1$, we have $1 > (1-\overline{K}_{\mathrm{C}} + \overline{p})^{1/2} > (1-\overline{K}_{\mathrm{C}} + \overline{p})$. Therefore the root $\breve{w}_- < \overline{p}$ lies outside the range of interest $w \in [\overline{p}, \overline{C}_{\mathrm{Cg}}(\overline{C}_{\mathrm{C}})]$ and the desired root is $\breve{w}_+ > \overline{p}$. For notational consistency we rename this value $\breve{w}_+ \stackrel{\text{def}}{=} \widehat{w}_0$. At $\widehat{w}_0$, the curve $y_{\max}(w)$ achieves a minimum $y$-value. It is not guaranteed that the point $(\widehat{w}_0, y_{\max}(\widehat{w}_0))$ lies in the region $\Gamma_{-+}$; in fact for the values in Table 1, $\overline{C}_{\mathrm{Cg}}(\overline{C}_{\mathrm{C}}) < \widehat{w}_0 < \overline{K}_{\mathrm{C}}$. Evaluated using parameter values from Table 1, the segment of the curve $y_{\max}(w)$ lying in $\Gamma_{-+}$ is plotted in Fig. 3.

We now consider the crossing point $(w_\Gamma, y_\Gamma)$ of the curves $y_{\max}(w)$ and $y_{n0}(w)$. From (26) and (D.37), $w_\Gamma$ satisfies

$$U_\Gamma^9(U_\Gamma - \Delta + 1) + \overline{s}^9(U_\Gamma - \Delta)/\delta = 0, \tag{D.38}$$

where $U_\Gamma = w_\Gamma - \overline{p}$, $\Delta = \overline{K}_{\mathrm{C}} - \overline{p}$.

**Lemma D.** *Equation (D.38) has a single solution $U_\Gamma \in [0,\Delta]$ (i.e. $w_\Gamma \in [\overline{p}, \overline{K}_C]$). In particular, $U_\Gamma \cong (1+\varepsilon)\overline{s}$ where $\varepsilon < 10^{-1}$.*

**Proof:** Let $h(U)$ denote the function

$$h(U) = U^9(U+1-\Delta) + \frac{\overline{s}^9(U-\Delta)}{\delta}.$$

We are only interested in the range $U \in [0,\Delta]$. Noting that

$$h(0) = -\frac{\overline{s}^9\Delta}{\delta} < 0, \qquad h(\Delta) = \Delta^9 > 0,$$

$$h'(U) = U^8[10U + 9(1-\Delta)] + \frac{\overline{s}^9}{\delta}, \qquad h''(U) = 9U^7[10U + 8(1-\Delta)] > 0,$$

we see that $h'(U) > 0$ on $[0,\Delta]$ and conclude that $h(U) = 0$ for a single $U_\Gamma \in [0,\Delta]$. Substituting $U_\Gamma \cong (1+\varepsilon)\overline{s}$ into (D.38), to first order in a Taylor expansion in $\varepsilon$ (assumed small) we find

$$\varepsilon = \frac{1-(1+\delta)(\overline{s}+1-\Delta)}{(1+10\delta)\overline{s} + 9\delta(1-\Delta)}.$$

Using parameter values from Table 1 we have $\varepsilon \approx 0.094$. Using a second order Taylor expansion yields $\varepsilon \approx 0.071$. ■

From Lemma D we conclude the curve $y_{\max}(w)$ crosses $\Gamma_{l0}$ at the single point $w_\Gamma$, $y_\Gamma$ where

$$\overline{p}+\overline{s} \lesssim w_\Gamma = \overline{p}+(1+\varepsilon)\overline{s} < \overline{C}_{\mathrm{Cg}}(\overline{C}_{\mathrm{C}}), \qquad y_\Gamma = \left[\frac{\overline{s}}{w_\Gamma - \overline{p}}\right]^3 = (1+\varepsilon)^{-3}.$$

This crossing point is also shown in Fig. 3.

The behavior of $W_2(y)$ depends on two cases, DI: $w_\Gamma \le w_*$ or DII: $w_\Gamma > w_*$.

**Case DI:** $w_\Gamma \le w_*$. We examine all possible values of $w$.

**DIa:** Let $w \in (\overline{p}, \widehat{w}_1)$. Since $\widetilde{F}(w,0) \to -\infty$, $\widetilde{F}(w,1) < 0$ and $\partial \widetilde{F}(w,y)/\partial y > 0$, we conclude $\widetilde{F}(w,y)|_{\Gamma_{-+}} < 0$ for $w \in (\overline{p}, \widehat{w}_1)$.

**DIb:** Let $w = \widehat{w}_1$. Since $\widetilde{F}(\widehat{w}_1, 0) \to -\infty$, $\widetilde{F}(\widehat{w}_1, 1) < 0$ and $\partial \widetilde{F}(w,y)/\partial y|_{\widehat{w}_1} > 0$ for $0 < y < 1$, we conclude $\widetilde{F}(\widehat{w}_1, y)|_{\Gamma_{-+}} < 0$.

**DIc:** $w \in (\widehat{w}_1, w_\Gamma)$. For any $w$ in this range, $\widetilde{F}(w,y)$ goes through a maximum value at $y_{\max}(w)$. While $\widetilde{F}(w,y) < 0$ near the boundaries $y = 0$ and $y = \min(1, y_{l0}(w))$, it is possible that $\widetilde{F}(w, y_{\max}(w)) \ge 0$. Thus for $w \in (\widehat{w}_1, w_\Gamma)$, $\widetilde{F}(w_\Gamma, y)|_{\Gamma_{-+}}$ may have: i) no real roots, ii) one real root $y(w) = y_{\max}(w)$, or iii) two real roots $y_1(w)$, $y_2(w)$ satisfying $0 < y_1(w) < y_{\max}(w) < y_2(w) < \min(1, y_{l0}(w))$.

**DId:** $w = w_\Gamma$. This case is similar to DIb with the upper boundary value being $y_{l0}(w_\Gamma)$. Thus $\widetilde{F}(w_\Gamma, y)|_{\Gamma_{-+}} < 0$.

**DIe:** $w \in (w_\Gamma, w_*)$. This case is similar to DIa with the upper boundary value being $y_{l0}(w)$. Thus $\widetilde{F}(w,y)|_{\Gamma_{-+}} < 0$.

**DIf:** $w = w_*$. $\widetilde{F}(w_*, y)|_{\Gamma_{-+}}$ has one real root at the point $(w_*, y_*)$.

**DIg:** $w \in (w_*, \overline{C}_{\mathrm{Cg}}(\overline{C}_{\mathrm{C}})]$. Here $\lim_{y\to 0^+} \widetilde{F}(w,y) \to -\infty$ and $\widetilde{F}(w, y_{l0}(w)) > 0$. Since $\partial \widetilde{F}(w,y)/\partial y > 0$ on $0 < y < y_{l0}(w)$, we conclude $\widetilde{F}(w,y)|_{\Gamma_{-+}}$ has a single real zero, $y_1(w)$, satisfying $0 < y_1(w) < y_{l0}(w)$.

Cases DIf and DIg show that $\widetilde{F}(w,y)|_{\Gamma_{-+}} = 0$ only on a curve segment traced out by the root $y_1(w)$ for $w \in [w_*, \overline{C}_{\mathrm{Cg}}(\overline{C}_{\mathrm{C}})]$. Since DIe states that $\widetilde{F}(w,y)|_{\Gamma_{-+}} \neq 0$ for $w \in (w_\Gamma, w_*)$, we conclude that: 1) the curve segment identified in DIf and DIg is uniquely the continuous curve segment $W_2(y)$ identified previously, and 2) case DIc can only support the conclusion $\widetilde{F}(w,y) < 0$ for $w \in (\widehat{w}_1, w_\Gamma)$. Combining the results of D0 and DI, we have verified that $W_2(y)$ connects $(w_*, y_*)$ to $(\overline{C}_{\mathrm{Cg}}(\overline{C}_{\mathrm{C}}), y_{\mathrm{F1}})$ and $W_2(y)$ is a continuous, one-to-one, onto map, $[w_*, \overline{C}_{\mathrm{Cg}}(\overline{C}_{\mathrm{C}})] \leftrightarrow [y_*, y_{\mathrm{F2}}]$. The form of the curve $W_2(y)$ for case DI is sketched in Fig. 3.

**Case DII:** $w_\Gamma > w_*$. As in case DI, we examine all possible values of $w$. As the arguments are generally similar to case DI, we shorten the presentation for each case.

**Cases DIIa** and **DIIb** are identical to DIa and DIb respectively.

**DIIc:** $w \in (\widehat{w}_1, w_*)$. For each $w$ in this range, $\widetilde{F}(w_\Gamma, y)|_{\Gamma_{-+}}$ can have: i) no real roots, ii) one real root $y_1(w) = y_{\max}(w)$, or iii) two real roots $y_1(w)$, $y_2(w)$ satisfying $0 \le y_1(w) < y_{\max}(w) \le y_2(w) < \min(1, y_{l0}(w))$.

**DIId:** $w = w_*$. As $\widetilde{F}(w_*, y_{\max}(w_*)) > \widetilde{F}(w_*, y_*) = 0$ then $\widetilde{F}(w_*, y)|_{\Gamma_{-+}}$ has exactly two real roots $y_1(w_*)$, $y_2(w_*)$ satisfying $0 < y_1(w_*) < y_{\max}(w_*) < y_2(w_*) = y_*$.

**DIIe:** $w \in (w_*, w_\Gamma)$. This case is similar to DIg. Thus $\widetilde{F}(w,y)|_{\Gamma_{-+}}$ has a single real zero $y_1(w)$ satisfying $0 < y_1(w) < y_{l0}(w)$.

**DIIf:** $w \in [w_\Gamma, \overline{C}_{\mathrm{Cg}}(\overline{C}_{\mathrm{C}})]$. The argument follows DIg; we conclude $\widetilde{F}(w,y)|_{\Gamma_{-+}}$ has a single real zero, $y_1(w)$, satisfying $0 < y_1(w) < y_{l0}(w)$.

The curve traced out by the root $y_1(w)$ in cases DIIa through DIIf must accord with the curve $W_2(y)$ on which $\widetilde{F}(w,y)|_{\Gamma_{-+}} = 0$. We therefore conclude that case DIIc must read:

**DIIc′** $w \in (\widehat{w}_1, w_*)$. There exists $w_m \in (\widehat{w}_1, w_*)$ such that: for any $w \in (\widehat{w}_1, w_m)$, $\widetilde{F}(w,y)|_{\Gamma_{-+}}$ has no real roots; for $w = w_m$, $\widetilde{F}(w,y)|_{\Gamma_{-+}}$ has one real root $y_m = y_{\max}(w_m)$; and for any $w \in (w_m, w_*)$, $\widetilde{F}(w,y)|_{\Gamma_{-+}}$ has two real roots $y_1(w)$, $y_2(w)$ satisfying $0 < y_1(w) < y_{\max}(w) < y_2(w) < y_*$. Combined with the results from cases D0, we have that on $[y_{\mathrm{F2}}, y_m]$, $W_2(y)$ strictly decreases in value from $\overline{C}_{\mathrm{Cg}}(\overline{C}_{\mathrm{C}})$ to $w_m$, and on $[y_m, y_*]$, $W_2(y)$ strictly increases in value from $w_m$ to $w_*$.The form for the curve $W_2(y)$ in DII and the point $(w_m, y_m)$ are illustrated in Fig. 3.

Finally we note that cases DIg and DIIf show the existence of a single root of $\widetilde{F}(w,y)|_{\Gamma_{-+}}$ on the boundary segment $w = \overline{C}_{\mathrm{Cg}}(\overline{C}_{\mathrm{C}})$, $y \in (0, y_{l0}(\overline{C}_{\mathrm{Cg}}(\overline{C}_{\mathrm{C}})))$. This root is the unique point $(\overline{C}_{\mathrm{Cg}}(\overline{C}_{\mathrm{C}}), y_{\mathrm{F2}})$ claimed in (D.33).

## Appendix E $\widetilde{F}(w,y)$ in $\Gamma_{--}$

We address the variation of the sign of $\tilde{F}(w,y)$ in $\Gamma_{--}$ by analyzing (23e). We start with the behavior of $\widetilde{F}(w,y)$ on the boundaries of $\Gamma_{--}$. From (23d) and restriction (24) we have demonstrated in the paper that $\widetilde{F}(w,y)|_{\Gamma_{\mathrm{g}0}} = \widetilde{F}(\overline{p}, y) < 0$. On the boundary $y = 0$, $w \in [0, \overline{p})$ we have $\lim_{y\to 0^+} \widetilde{F}(w,y) \to -\infty$. From (23e) it is straightforward to check that $\widetilde{F}(0,y) = \overline{K}_{\mathrm{C}}\overline{C}_{\mathrm{C}}\overline{Q} > 0$. The behavior of $\widetilde{F}(w,y)|_{\Gamma_{--}}$ on the boundary $y = 1$, $w \in [0, \overline{p}]$ can be determined by examining the behavior of $\widetilde{F}(w,1)|_{\Gamma_{--}}$ with respect to w. From (23e) we can write

$$\widetilde{F}(w,y) = \overline{K}_{\mathrm{C}}\overline{C}_{\mathrm{C}}\overline{Q} + e_1(y)w + e_2(y)w^2 + \overline{\Lambda}_l w^3, \tag{E.40}$$

where $e_1(y) = d_1(y)|_{g(y)=0}$ and $e_2(y) = d_2(y)|_{g(y)=0}$. From (E.40) we have the following properties

$$\lim_{w\to-\infty} \widetilde{F}(w,1) \to -\infty, \qquad \widetilde{F}(0,1) > 0,$$
$$\widetilde{F}(\overline{p},1) < 0 \text{ by (24)}, \qquad \lim_{w\to\infty} \widetilde{F}(w,1) \to \infty. \tag{E.41}$$

Equations (E.40) and (E.41) imply that $\widetilde{F}(w,1)$ has three real roots, $w_1$, $w_2$, $w_3$, satisfying $w_1 < 0 < w_2 < \overline{p} < w_3$. Let $w_2 \overset{\text{def}}{=} w_{F3}$. Then we have

$$\widetilde{F}(w,1) > 0, \quad w \in [0, w_{F_3}), \qquad \widetilde{F}(w,1) < 0, \quad w \in (w_{F3}, \overline{p}]. \tag{E.42}$$

With the behavior of $\widetilde{F}(w,y)|_{\Gamma_{--}}$ determined on the boundaries, we now consider its behavior in the interior. From (23e), $\widetilde{F}(w,y)$ can be written

$$\widetilde{F}(w,y) = E_0(w) + E_1(w)y^{-1/3}, \tag{E.43}$$

where $E_1(w) = -w\overline{\Lambda}_l\overline{p}(1 - \overline{K}_C + w) < 0$. The first two partials of $\widetilde{F}(w,y)$ with respect to $y$ are

$$\frac{\partial\widetilde{F}(w,y)}{\partial y} = -\frac{1}{3}E_1(w)y^{-4/3} > 0, \qquad \frac{\partial^2\widetilde{F}(w,y)}{\partial y^2} = \frac{4}{9}E_1(w)y^{-7/3} < 0. \tag{E.44}$$

From (E.42) and (E.44) we conclude the following.
i) For each fixed $w \in (0, w_{F3}]$, $\widetilde{F}(w,y) = 0$ at exactly one value $y = Y_3(w)$, with $Y_3(w_{F3}) = 1$. For $y \in (0, Y_3(w))$, $\widetilde{F}(w,y) < 0$ and for $y \in (Y_3(w), 1)$, $\widetilde{F}(w,y) > 0$.
ii) For each fixed $w \in (w_{F3}, \overline{p}]$, $\widetilde{F}(w,y) < 0$.
From i) and ii) we have the existence of the curve $Y_3(w)$ along which $\widetilde{F}(w, Y_3(w)) = 0$. From (E.43), $Y_3(w)$ has the explicit form $Y_3(w) = (-E_1(w)/E_0(w))^3$ satisfying $\lim_{w\to0^+} Y_3(w) = 0$.

As $\widetilde{F}(w, Y_3(w)) = 0$, we can compute an expression for the slope of $Y_3(w)$ from (E.43),

$$\begin{aligned}
0 &= \frac{dE_0(w)}{dw} + \frac{dE_1(w)}{dw}Y_3(w)^{-1/3} - \frac{1}{3}E_1(w)Y_3(w)^{-4/3}\left(\frac{dY_3(w)}{dw}\right), \\
&= \left.\frac{\partial\widetilde{F}(w,y)}{\partial w}\right|_{Y_3(w)} + \left.\frac{\partial\widetilde{F}(w,y)}{\partial y}\right|_{Y_3(w)} \frac{dY_3(w)}{dw}, \\
&\to \frac{dY_3(w)}{dw} = -\left.\frac{\partial\widetilde{F}(w,y)}{\partial w}\right|_{Y_3(w)} \Bigg/ \left.\frac{\partial\widetilde{F}(w,y)}{\partial y}\right|_{Y_3(w)}.
\end{aligned} \tag{E.45}$$

From i) we know that $Y_3(w)$ maps the interval $w \in (0, w_{F3}]$ onto $y \in (0,1]$ in a one-to-one fashion. Therefore $Y_3(w)$ implicitly defines the inverse curve $W_3(y)$ which maps $y \in (0,1]$ onto $w \in (0, w_{F3}]$ in a one-to-one fashion with $\lim_{y\to0^+} W_3(y) = 0$ and $W_3(1) = w_{F3}$. As $W_3(y)$ is the only curve on which $\widetilde{F}(w,y)|_{\Gamma_{--}} = 0$, for each fixed $y \in (0,1]$ we have $\widetilde{F}(w,y) > 0$ for $w \in (0, W_3(y))$, and $\widetilde{F}(w,y) < 0$ for $w \in (W_3(y), \overline{p}]$. Thus we conclude $\partial\widetilde{F}(w,y)/\partial w|_{Y_3(w)} < 0$. By (E.44) we know $\partial\widetilde{F}(w,y)/\partial y|_{Y_3(w)} > 0$. Applied to (E.45), these observations lead to the conclusion, $dY_3(w)/dw > 0$. The curve $W_3(y)$ is sketched in Fig. 3.

## Appendix F Initial conditions for gas bubble formation

As noted in the discussion of solution trajectories in the paper, initial bubble size is determined by microscopic, non-linear dynamics at a nucleation site. To mimic this, we set the initial bubble radius equal to the radius value of the inlet. Thus,

$$y_0 = (a/\mathrm{R_{pore}})^3. \tag{F.46}$$

To determine initial bubble $CO_2$ concentration, we assume that the pressure in the liquid phase does not change at the moment of bubble formation. From (14′) this assumption gives $\overline{p}_l^{\mathrm{sp}} = \overline{p}_\mathrm{g} - \overline{p}_c = w_0 - \bar{s}y_0^{-1/3}$. Using (2) we derive a value for $w_0$,

$$w_0 = \overline{p} + \bar{s}y_0^{-\frac{1}{3}} + \overline{Q}/\overline{\Lambda}_l = w_{\iota0}(y_0) + \overline{Q}/\overline{\Lambda}_l. \tag{F.47}$$

Finally, we impose mass conservation at the time of bubble formation; the loss of $CO_2$ in the liquid phase must equal the amount of $CO_2$ in the gas bubble. (This is consistent with our previous assumption that phase changes occur faster than flow rates.) Using the two-phase flow relation (13) between $\overline{C}_{Cl}$ and $w = \overline{C}_{Cg}$, $CO_2$ conservation gives

$$\overline{C}_{Cl}^{sp}(t_b) - \frac{w_0}{\overline{K}_C - w_0}(1 - y_0) = w_0 y_0, \tag{F.48a}$$

where $\overline{C}_{Cl}^{sp}(t_b)$ denotes the $CO_2$ concentration in the liquid just prior to bubble formation at time $t_b$. Using (2), (F.48a) can be used to solve for $t_b$,

$$t_b = -\frac{1}{\overline{Q}} \ln\left\{1 - \frac{1}{\overline{C}_C}\left(\frac{w_0}{\overline{K}_C - w_0}\right)[1 - (1 + w_0 - \overline{K}_C)y_0]\right\}. \tag{F.48b}$$

## Appendix G

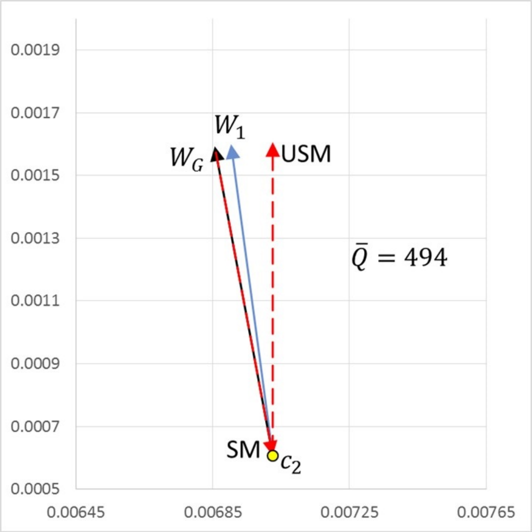


**Figure G.** Properly scaled figure showing directions at the critical point $c_2$. The red arrow labeled USM corresponds to the direction of the unstable manifold and the red arrow labeled SM is the direction of the stable manifold. Also noted (black, blue arrows) are the directions of the curves and $W_G$ and $W_1$ at $c_2$. The direction of $W_G$ is almost antiparallel to that of the stable manifold.

## Appendix H

**Assumptions used in analysis of the dynamical system** (18′)

The liquid phase is incompressible

The concentration of water in the liquid phase, $C_W$, is constant

The outlet reservoir remains pure water

Phase changes occur more rapidly than fluid transport

The liquid phase is perfectly wetting

Any gas phase forming in a pore occurs as a single bubble

The gas phase acts as an ideal gas

The thermodynamic values $\overline{K}_H(T)$ and $\overline{K}_C(T)$ satisfy $\overline{K}_H(T)/\overline{K}_C(T) \approx 0$ implying that the concentration of $H_2O$ in the gas phase is negligible, $\overline{C}_{Hg}(w) \approx 0$

$w \gg \overline{K}_H(T) = O(10^{-5})$

The flow rate, $\overline{Q}$, is restricted to the range given by equation (24)

Bubble initial conditions: initial bubble radius equals inlet radius liquid pressure does not change at moment of bubble formation

$4\overline{K}_{\mathrm{C}}/(1+\overline{C}_{\mathrm{C}}+\overline{K}_{\mathrm{C}})^2 \ll 1$

$\widetilde{F}(\overline{C}_{\mathrm{Cg}}(\overline{C}_{\mathrm{C}}),y)$ has no zero on the interval $y \in (y_{G2}, y_*)$ when $y_* > y_{G2}$

$4\bar{s}\delta \ll (\overline{K}_{\mathrm{C}}+\overline{p}+\bar{s}\delta)^2$ where $\delta = \overline{\Lambda}_l/(9\overline{\Lambda}_{\mathrm{g}})$
